# An anatomically detailed arterial-venous network model. Cerebral and coronary circulation

**DOI:** 10.3389/fphys.2023.1162391

**Published:** 2023-06-26

**Authors:** Lucas O. Müller, Sansuke M. Watanabe, Eleuterio F. Toro, Raúl A. Feijóo, Pablo J. Blanco

**Affiliations:** ^1^ Department of Mathematics, University of Trento, Trento, Italy; ^2^ Federal University of Agreste de Pernambuco, UFAPE, Garanhuns, Brazil; ^3^ National Institute of Science and Technology in Medicine Assisted by Scientific Computing, INCT-MACC, Petrópolis, Brazil; ^4^ Laboratory of Applied Mathematics, Department of Civil, Environmental and Mechanical Engineering, University of Trento, Trento, Italy; ^5^ National Laboratory for Scientific Computing, LNCC/MCTI, Petrópolis, Brazil

**Keywords:** haemodynamics, wave propagation, arterial-venous system, cardiovascular diseases, brain circulation, coronary circulation

## Abstract

In recent years, several works have addressed the problem of modeling blood flow phenomena in veins, as a response to increasing interest in modeling pathological conditions occurring in the venous network and their connection with the rest of the circulatory system. In this context, one-dimensional models have proven to be extremely efficient in delivering predictions in agreement with *in-vivo* observations. Pursuing the increase of anatomical accuracy and its connection to physiological principles in haemodynamics simulations, the main aim of this work is to describe a novel closed-loop Anatomically-Detailed Arterial-Venous Network (ADAVN) model. An extremely refined description of the arterial network consisting of 2,185 arterial vessels is coupled to a novel venous network featuring high level of anatomical detail in cerebral and coronary vascular territories. The entire venous network comprises 189 venous vessels, 79 of which drain the brain and 14 are coronary veins. Fundamental physiological mechanisms accounting for the interaction of brain blood flow with the cerebro-spinal fluid and of the coronary circulation with the cardiac mechanics are considered. Several issues related to the coupling of arterial and venous vessels at the microcirculation level are discussed in detail. Numerical simulations are compared to patient records published in the literature to show the descriptive capabilities of the model. Furthermore, a local sensitivity analysis is performed, evidencing the high impact of the venous circulation on main cardiovascular variables.

## 1 Introduction

Blood flow modeling and simulation in the cardiovascular system amounts to tackle several challenges across disciplines: mathematical complexity, connection to clinical concepts, difficulty of model setting, viability of model validation/verification, quantity of input data required by models and, eventually, computational cost. In this context, one-dimensional (1D) modeling offers an approach based on basic physical principles, a relatively low model complexity (from the mathematical point of view and from the input data perspective) and proximity between model ingredients and quantifiable/verifiable variables in clinical practice, while retaining excellent predictive and descriptive capabilities in terms of behavior of blood pressure and flow rate along networks of vessels.

Feasibility and capacity of 1D models in predicting haemodynamic features have been reported at the different scales of blood circulation and for many physiological settings. In fact, the use of 1D models has extended from its application to studying haemodynamics in large arteries, as proposed in (Noordergraaf et al., 1963; Avolio, 1980), just to mention a few seminal works in the field, to understanding waveform composition mechanisms (Alastruey et al., 2014; Willemet and Alastruey, 2015), to gaining insight about the impact of cardiovascular diseases (Stergiopulos et al., 1992; Alastruey et al., 2007; Liang et al., 2009; Willemet et al., 2013), to understanding the connection to microcirculation networks (Perdikaris et al., 2015), to providing boundary conditions to 3D blood flow models (Urquiza et al., 2006; Blanco et al., 2009; Blanco et al., 2010; Malossi et al., 2013; Perdikaris et al., 2016) and, more recently, to studying haemodynamics also in large veins (Liang and Liu, 2005; Müller and Toro, 2014a; Müller and Toro, 2014b; Mynard and Smolich, 2015; Celant et al., 2021; Toro et al., 2022). Yet, there is plenty of room for the effective application of 1D blood flow models to study arteriolar and capillary networks. In this regard, available computational tools are able to provide, with high detail, morphometrically significant vascular networks on top of which 1D models can be set (Karch et al., 1999; Blanco et al., 2013). Up to date, mainly 0D models have been employed in addressing problems at this scale (Reichold et al., 2009; Linninger et al., 2013), while the use of 1D models is limited (Lee and Smith, 2008; Pan et al., 2014).

Another fundamental issue that boosted the spread of contributions using 1D models is that more mature and computationally efficient mathematical and numerical tools are currently available. This has allowed the community to address increasingly complex 1D simulations, ranging from the solution of blood flow in extremely large networks of vessels (Blanco et al., 2015) to the estimation of model parameters from *in-vivo* data (Lombardi, 2014), as well as the quantification of model uncertainties (Chen et al., 2013). Importantly, the resurface of 1D modeling has been accompanied by solid *in-vitro* validations (Matthys et al., 2007; Bessems et al., 2008; Alastruey et al., 2011), *in-vivo* verifications (Stettler et al., 1981; Olufsen et al., 2000; Reymond et al., 2009; Reymond et al., 2011), *in silico* validations (Grinberg et al., 2011; Xiao et al., 2014) and more recently methodological head-to-head comparisons (Boileau et al., 2015).

Recently, we have developed the ADAN (Anatomically Detailed Arterial Network) model (Blanco et al., 2015) which provides a cutting-edge 1D modeling framework to simulate complex haemodynamics scenarios. Because of its extreme anatomical detail, one of the main features of the ADAN model is that it naturally allows to establish the connection between the vascular anatomy of large arteries and the distributed arteriolar networks through the concept of vascular territories (Blanco et al., 2012; Blanco et al., 2014), making possible to widen the range of physiological and pathophysiological scenarios addressable by the model. Because of this, the ADAN model, when properly coupled to arteriolar networks, is capable of providing a direct pathway to assess coupled arterial-arteriolar haemodynamics. As example of the potentialities of the model, we mention the study of steal phenomena reported in (Blanco et al., 2016a), the study of the role of hypertension in the mechanisms underlying small vessel disease reported in (Blanco et al., 2016b), as modeling support of the ambibaric brain hypothesis (Hachinski and Østergaard, 2021).

The goal of the present work is to describe the first stage of the development of a novel arterial-venous model featuring high anatomical detail to perform 1D blood flow simulations. Hereafter, this model will be referred to as Anatomically Detailed Arterial-Venous Network (ADAVN) model. To the best of our knowledge, the ADAVN model is the most complex arterial-venous closed loop model present in the literature with a one-dimensional description of systemic arteries and veins. The arterial network of the ADAVN model is that of the ADAN model, and it is coupled to a novel venous network that is constructed following equivalent premises. In this first stage of the model construction, the venous network in the ADAVN model features high detail in the vascular anatomy of the cerebral and coronary circulations. The cerebral venous network is similar to the one built in (Müller and Toro, 2014a; Müller and Toro, 2014b) in terms of vessels included in the model for this venous district. This similarity is dictated by the aim of being able to reproduce the interplay of intracranial pressure and cerebral venous dynamics described in (Müller and Toro, 2014a) and further explored in (Toro et al., 2022), with the current version of ADAVN. This requirement is also reflected in the adopted strategy to describe cerebral venous haemodynamics by including a different mechanical parametrization for cerebral veins and dural sinuses, as well as in the choice of connecting cerebral veins to dural sinuses via Starling resistors, as done in (Müller and Toro, 2014a; Toro et al., 2022). The venous network is composed by 189 veins, featuring 79 venous vessels draining cerebral vascular territories and 14 vessels draining coronary territories. We refer to this version of the model as a first model development stage because our intention is that of incrementally adding anatomical and functional complexity in future works.

The mathematical model corresponds to the classical 1D blood flow equations for both arterial and venous vessels with standard coupling conditions at junctions. The peripheral coupling between the arterial and venous networks is achieved using lumped Windkessel models. A lumped model of the heart chambers and pulmonary circulation is employed to close the loop. The setting of model parameters, specifically the behavior of the vessel wall and the parameters of peripheral (terminal) models, will be thoroughly discussed. Particular attention will be given to the methodology used for the management of arterial supply to and venous drainage from vascular territories.

As with the ADAN model, the rationale to develop a highly detailed model of the venous network is the need to assemble, in an incremental manner, a haemodynamic modeling framework, capable to accommodate refined anatomical data, basic principles of human physiology and fundamental knowledge related to pathophysiological conditions, to perform modeling-based research on cardiovascular physiology. This includes the possibility to gain insight into physiological mechanisms of blood circulation as well as to study abnormal conditions encountered in disease.

One of the major motivations to improve the description of the venous vascular anatomy is that, due to the fact that blood pressure in this system is low, if compared to the arterial counterpart, blood drainage is performed through an extremely complex arrangement of vessels that constitutes a highly collateralized network. These collateral circuits play fundamental functional roles in many situations, both in health and disease. Blood drainage is of the uttermost importance in organs such as the brain and the heart. Moreover, particularly to these organs is the fact that veins are exposed to complex environments, such as the one established by the interactions with the cerebrospinal fluid (CSF) and by the impact of the contraction of the heart, respectively. It can therefore be appreciated that a detailed vascular model promotes and facilitates a framework in which the physiological interaction with other systems of the human body are conceptually natural and more straightforward to be integrated. As well, there are several other pathological conditions that trigger the interest in the development of detailed computational models of the venous system. Some of them are: extracranial venous strictures (Zamboni et al., 2008); arteriovenous malformations (Chen et al., 2020); orthostatic stress intolerance (Stewart, 2013); varicose veins (Gawas et al., 2022) and portal hypertension (Mauro and Gadano, 2020).

This work is organized as follows.Section 2 presents ADAVN topology (Section 2.1), the connection between arterial and venous circulations (Section 2.1.3), the mathematical models and numerical methods used for the construction of the present model (Sections 2.2, 2.3) and model parameters (Section 2.4). Next, in Section 3 we present results in terms of model performance with respect to major cardiac and cardiovascular indexes, as well as a local sensitivity analysis. This section is followed by the discussion of results and considerations about possible future research (Section 4).

## 2 Materials and methods

### 2.1 Arterial-venous network topology

In this section we describe the topology of the vessel network for both the arterial system and the venous system. Detailed information of model connectivity is provided in the Supplementary File adavn_vessels.csv.

#### 2.1.1 Arterial system

The arterial system considered here is the one corresponding to the ADAN model (Blanco et al., 2014; Blanco et al., 2015), with subsequent improvements reported in (Blanco et al., 2016a). The ADAN model was built using data extracted from classical anatomical textbooks (Dauber, 2007; Netter, 2011) and features an average male vascular anatomy. This procedure consisted in manually translating the 2D pictures of vascular circuits featured in (Netter, 2011) into the 3D space, over a digital dataset of a human skeleton as scaffold. Arterial vessels listed in (Hood, 1968), with a well-established name according to the anatomical terminology were included in the model.

Arterial vessels were outlined in 3D space using cubic splines in software Autodesk 3ds Max (version 2010) with the aid of a human skeleton as scaffold. Almost all arteries with a name according to the anatomical terminology were included in the model. This yields 1,598 named arteries. In addition, the model contains perforator vessels which supply blood to peripheral regions. The ADAN model incorporates 28 specific organs (i.e., kidneys, liver, heart, *etc.*) and 116 vascular territories, which include distributed organs (muscles, skin, *etc.*).

**FIGURE 1:**
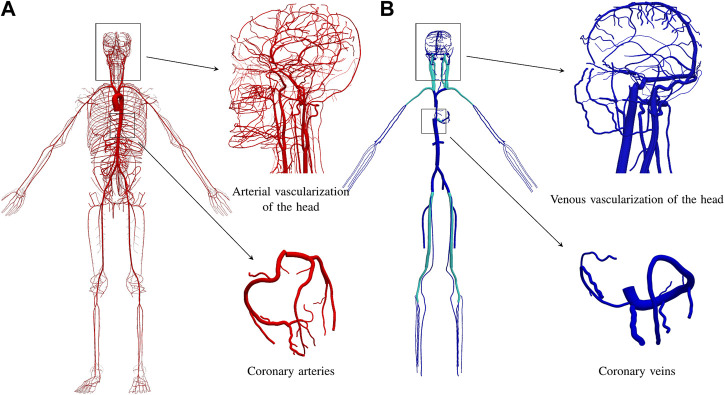
Vascular anatomy of the ADAVN model. **(A)** arterial vascular anatomy with over 2,185 vessels. **(B)** venous vascular anatomy with 189 vessels. Insets show details of the circulation circuits in the head and in the heart. Veins colored in cyan contain a venous valve at its proximal extremity.

Figure 1A presents the arterial network of the ADAVN model. The coronary network consists of 23 arteries, for an average left dominant vascular topology. A complete circle of Willis is considered in the brain circulation, and the intracranial network contains 162 arteries.

#### 2.1.2 Venous system

The venous system of the ADAVN model includes the largest veins in the human body. Overall, the venous network is composed by 189 veins, which drain blood from 66 vascular regions, see Section 2.2.4. Since the venous system is not as detailed as the arterial one, most of the peripheral venous vessels drain blood from more than one vascular territory and/or specific organ, as defined in the ADAN model. The procedure for the delineation of the venous system followed the same procedure as explained for the arterial system. That is, heac venous vessel was manually mapped from the anatomical textbooks to the 3D space on top of the skeleton scaffold. However, in this first stage only cerebral and coronary venous vessels were fully included in the model, while the rest of the venous system was represented through the major vessels.

The lower limbs are drained by the great saphenous veins (GSVs) as well as the anterior and posterior tibial veins, which conduct blood to the popliteal veins and then to the femoral veins (FVs).

The external iliac veins (EIVs) gather blood from the GSVs and the FVs, converging with the internal iliac veins to the common iliac veins, and then to the inferior vena cavae (IVCs). The IVCs also carry blood from the splanchnic circulation towards the right atrium.

The upper limbs are drained by the radial, ulnar and anterior interosseous veins. These vessels converge to the brachial veins, and then to the axillary vein and the subclavian veins (SVs). The SVs together with the external jugular vein (EJVs), the internal jugular vein (IJVs) and the vertebral vein (VV)s carry blood to the brachiocephalic veins (BrVs). The left BrV also collects blood from the thyroidal territories through the inferior thyroid vein.

The EJVs drain the temporal regions, through the superficial temporal and posterior auricular veins. Also, the EJVs drain facial territories through the retromandibular veins, collecting blood from the facial vein (FVs) and deep facial veisn. Moreover, the FV anastomose to the IJVs through the common facial veins.

Figure 1B displays the venous network of the ADAVN model. In the following we describe the components of the venous system which have been characterized in detail in the current stage of the ADAVN model. Veins shown in cyan correspond to vessels that comprise a valve at its proximal extremity.

Cerebral veins have the role of draining the blood from the brain effectively. The ADAVN model contains 58 cerebral veins, among which we can distinguish the cortical veins, deep veins such as internal cerebral and Rosenthal veins and opthalmic veins.

The superior sagittal sinus (SSS) collects blood from the medial and lateral parts of the cortex through the (occipital, parietal and prefrontal) superior cerebral veins.

The inferior sagittal sinus (ISS) runs over the corpus callosum and drains blood from the central part of the brain. The straight sinus (StS) drains blood from the ISS and from the vein of Galen, which collects blood from the deep parts of the brain, through the internal cerebral veins and through the basal veins of Rosenthal. The confluence of sinuses (CoS) provides a connection among the SSS, the StS, the occipital sinus (OS) and the transverse sinus (TS). After collecting most of the blood coming from the SSS and the StS, the TS drains blood from the occipital and temporal superficial parts of the brain, through the corresponding inferior cerebral veins in that region. Then, towards the anterior part of the brain the TS connects to the sigmoid sinus (SiS) and superior petrosal sinus, which in turn connect to the inferior petrosal sinus (IPS), the cavernous and the posterior intracavernous sinuses. At that point, the basilar plexus is also connected, and provides a direct pathway to the confluence of sinuses through the marginal sinus and the OS.

The Trolard vein and the vein of Labbe provide corresponding shunts between the SSS, the TS and the superficial middle cerebral vein, which, in turn, is connected to the sphenoparietal sinus and, after collecting blood from the opthalmic veins, anastomoses to the cavernous sinus.

IJVs collect blood from the brain, draining from the SiS and the IPS, and from face and neck, finally arriving at the BrV.

The lateral anterior condylar vein connects the IJV with the CoS through the occipital vein, and towards the heart with the suboccipital sinus, from which the vertebral vein and the deep cervical brain branch as important collateral pathways for the blood to be drained towards the BrV.

**FIGURE 2:**
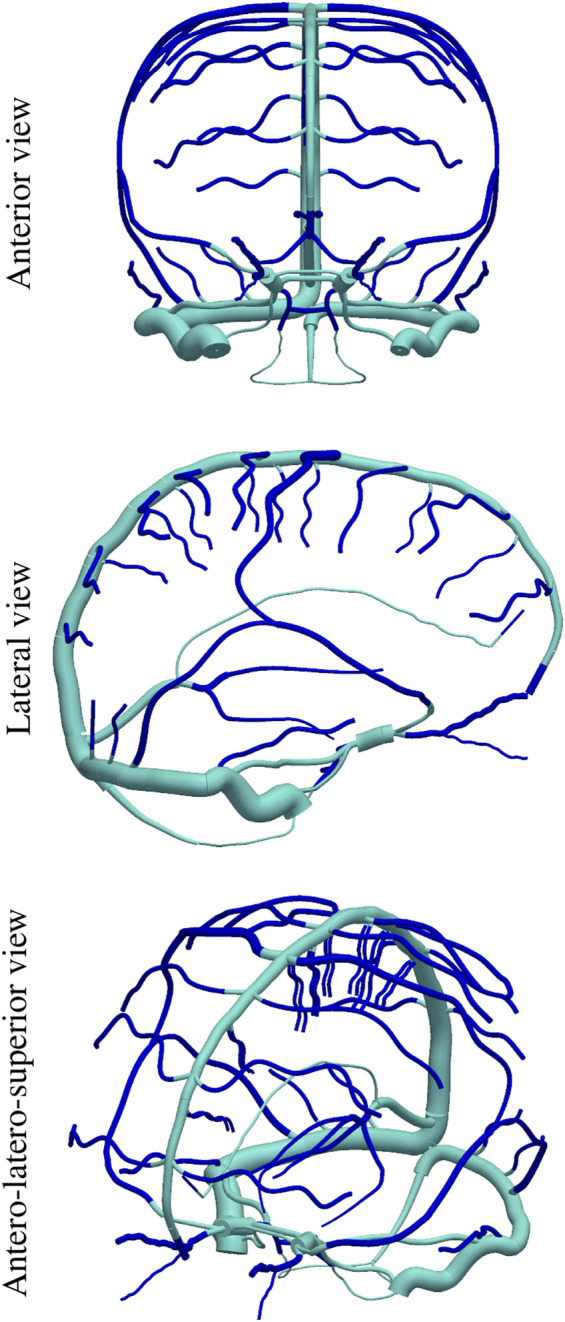
Position of Starling-resistor elements in the cerebral venous network, dividing the vascular network into intracranial cerebral veins (dark blue) and extracranial dural sinuses (light blue).

An additional ingredient of the present model is the existence of Starling-like elements which are able to account for the venous waterfall effect between dural sinuses and cerebral veins, wherein, when a portion of the vessel collapses the flow becomes independent of the central venous pressure (Müller and Toro, 2014a). This ingredient targets the hypothesis that establishes that CBF is ruled by the difference between arterial blood pressure and intracranial pressure. Figure 2 features the (color change) interfaces at which Starling elements are placed in the ADAVN model.

Coronary veins drain blood from the heart directly to the right atrium. There are 13 coronary veins, and the coronary sinus. The coronary sinus collects blood from the small cardiac vein (SCV), the posterior interventricular vein, the left ventricular vein, the oblique vein of left atrium and the great cardiac vein (GCV). The SCV drains blood from smaller veins of the right heart such as the anterior vein of right ventricle and other right (marginal, atrial and ventricular) veins. In turn, the tributaries to the GCV are the posterior vein of left ventricle, the anterior interventricular vein and the left atrial and marginal veins.

#### 2.1.3 Arterial-venous connectivity

The criteria and hypotheses to define the connectivity between arterial and venous peripheral beds were the following:

• each terminal artery is connected to an arteriolar bed, which can connect to multiple veins;
• the same is valid for each terminal vein, which is connected to a venular bed, which can receive blood from multiple arteries;
• connectivity was established according to existing knowledge on tributary/emissary vessels for vascular territories in the brain circulation [see for example, (Lang, 1995)] and in the coronary circulation [see for example, (Hood, 1968)], whenever available. In other cases connectivity was based on proximity of terminal arteries and terminal veins;
• the connection between arteriolar and venular beds is modeled through a purely resistive element to represent the capillary resistance.

**FIGURE 3:**
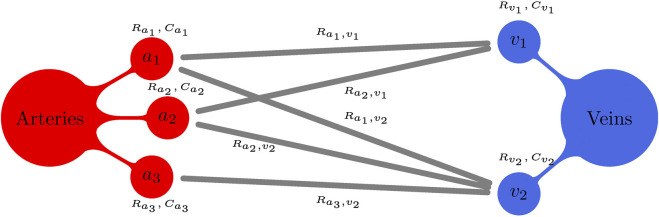
Schematic illustration for a generic peripheral circulation model. Terminal arteries *a*_*i*_, *i* = 1, 2, 3, give rise to three arteriolar/capillary compartments (with corresponding compliances $C_{a_{i}}$ and proximal resistances $R_{a_{i}}$ ). On the other hand, terminal veins *v*_*j*_, *j* = 1, 2, define two venous compartments (with corresponding compliances $C_{v_{j}}$ and proximal resistances $R_{v_{j}}$ ). Moreover, arterio-venous connections define arterio-venous resistances $R_{a_{i},v_{j}}$ .

Consider the connectivity model illustrated in Figure 3 with three terminal arteriolar districts (*a*_1_ *a*_2_, *a*_3_) and two terminal venular districts (*v*_1_ and *v*_2_), where we have:

• arteriolar bed *a*_1_ is a tributary to venular beds *v*_1_ and *v*_2_, with corresponding capillary resistances $R_{a_{1},v_{1}}$ and $R_{a_{1},v_{2}}$ ;
• arteriolar bed *a*_2_ is a tributary to venular beds *v*_1_ and *v*_2_, with corresponding capillary resistances $R_{a_{2},v_{1}}$ and $R_{a_{2},v_{2}}$ ;
• arteriolar bed *a*_3_ is a tributary to venular bed *v*_2_, with corresponding capillary resistance $R_{a_{3},v_{2}}$ .

Consequently, in this example

• venular bed *v*_1_ is an emissary from arteriolar beds *a*_1_ and *a*_2_;
• venular bed *v*_2_ is an emissary from arteriolar beds *a*_1_, *a*_2_ and *a*_3_.

The definition of the connectivity pattern for all terminal arteries and all venous vessels is provided in full detail in the Supplementary File adavn_vessels.csv. Specifically, in that file, the reader will find the description of the terminal arteries and veins, and their corresponding vascular districts, as well as the connectivity among them.

### 2.2 Mathematical model

This section is devoted to a detailed description of the partial and ordinary differential equations for all the components present in the model.

#### 2.2.1 Blood flow in compliant vessels

Classical one-dimensional blood flow equations (Hughes, 1974) are used to model the evolution of lumen area *A*, flow rate *q* and pressure *p* in the space-time domain, namely,

$$\left\{\begin{array}{cc} & \partial_{t}A+\partial_{x}q=0,\\ & \partial_{t}q+\partial_{x}\left(\frac{q^{2}}{A}\right)+\frac{A}{\rho }\partial_{x}p=-\frac{f}{\rho }, \end{array}\right.$$ (1)

where $f\left(x,t\right)=8\pi \mu \frac{q}{A}$ is the friction force per unit length of the tube, for a Poiseuille velocity profile, *μ* is the fluid viscosity and *ρ* is the fluid density. The first equation in (Eq. 1) represents mass conservation and the second one describes the balance of momentum. The relation between pressure and wall strain and strain rate is taken as follows

$$p\left(x,t\right)=\;p_{\text{ext}}\left(x,t\right)+p_{\text{tm}}\left(x,t\right).$$ (2)

Here, *p*_ext_(*x*, *t*) accounts for external pressure exerted by tissues or extravascular fluids on the vessel, while *p*_tm_ represents the transmural pressure, i.e., the pressure that is effectively being supported by stress in the vessel’s wall. In this work *p*_tm_ is different for arteries and veins. For arteries we consider the tube law previously used in (Blanco et al., 2015), namely,

$$p_{\text{tm}}^{\text{art}}=\frac{\pi R_{0}h_{0}}{A}\left[E_{e}\varepsilon +E_{c}\epsilon_{r}\ln\left(e^{\chi }+1\right)+\frac{K_{m}^{\text{art}}}{2\sqrt{AA_{0}}}\frac{\partial A}{\partial t}\right]+p_{0}^{\text{art}},$$ (3)

where *R*_0_ = *R*_0_(*x*) is the vessel radius at reference state $p_{\text{tm}}^{\text{art}}=p_{0}^{\text{art}}$ . The same is valid for cross-section area *A*_0_ = *A*_0_(*x*) and vessel wall thickness *h*_0_ = *h*_0_(*x*). *E*_*e*_ = *E*_*e*_(*x*) and *E*_*c*_ = *E*_*c*_(*x*) are the effective Young modulus of the elastin and collagen fibers, respectively, whereas *K*_*m*_ is the effective viscoelastic parameter. Furthermore, *χ* = *χ*(*A*, *x*) is

$$\chi =\frac{\varepsilon -\varepsilon_{0}}{\epsilon_{r}},$$ (4)

where *ɛ*_0_ = *ɛ*_0_(*A*_0_) is the deformation state for which 50% of collagen fibers have been activated, *ϵ*_*r*_ = *ϵ*_*r*_(*A*_0_) is the standard deviation of the fiber activation state distribution and *ɛ* = *ɛ*(*A*, *A*_0_) is the current deformation state, given by

$$\varepsilon =\sqrt{\frac{A}{A_{0}}}-1.$$ (5)

Tube law (Eq. 3) derives from a mixture theory approach that accounts for the different components of the arterial wall and their respective mechanical properties (Urquiza et al., 1995).

For veins we use a tube law proposed for collapsible tubes and previously used in models comprising a 1D description of the venous system (Müller and Toro, 2014a; Müller and Toro, 2014b; Toro et al., 2022). In particular, we use

$$p_{\text{tm}}^{\text{ven}}=K\left[{\left(\frac{A}{A_{0}}\right)}^{m}-{\left(\frac{A}{A_{0}}\right)}^{n}\right]+\frac{K_{m}}{A_{0}\sqrt{A}}\frac{\partial A}{\partial t}+p_{0}^{\text{ven}},$$ (6)

where *K* is the effective stiffness of the vessel’s wall, while *m* = 10 and *n* = −3/2 are coefficients responsible for the description of passive stiffening (*m*) and collapse (*n*).

At vessel junctions/bifurcations conservation of mass and energy are enforced by

$$\overset{N_{P}}{\underset{k=1}{\sum }}g_{k}\;q_{k}=0,$$ (7)

$$p_{1}+\frac{1}{2}\rho u_{1}^{2}-p_{k}-\frac{1}{2}\rho u_{k}^{2}=0,\;\;\;k=2,\ldots ,N_{P},$$ (8)

where *N*_*P*_ is the number of vessels converging at a junction and *g*_*k*_ = 1 if the *k*-vessel shares an outlet node with the junction and *g*_*k*_ = −1 if the shared node is the inlet one, according to the local system of reference within each vessel. Eq. 7 enforces mass conservation by requiring that no net mass change takes place at a junction/bifurcation node, while (Eq. 8) imposes total pressure continuity. Enforcing both conditions results in energy conservation, since the flux of energy is equal to the product of flow rate *q* and total pressure $p+\frac{1}{2}\rho u^{2}$ .

#### 2.2.2 Heart chambers and valves

The heart and its four valves are modeled as proposed in (Mynard, 2011; Mynard et al., 2012). The chamber model is based on experimental data that showed how the chamber pressure-volume relation can be modeled as an elastic compartment with prescribed time-varying elastance (Suga et al., 1973). In this work chamber pressure is a function of a time-varying elastance and of multiple interactions between chambers. In particular, chamber pressure is given by

$$p_{\alpha }=p_{\text{pc}}+E_{\text{nat},\alpha }\left(V_{\alpha }-V_{0,\alpha }\right)-R_{\text{S},\alpha }q_{\text{out},\alpha }+\frac{E_{\text{nat},\alpha }}{E_{\text{sep},\alpha }}p_{\text{CL},\alpha },$$ (9)

with *α* ∈ {RA, RV, LA, LV}. Here *p*_pc_ is the pericardial pressure, *V*_0,*α*_ is the reference chamber volume, *V*_*α*_ is the current chamber volume, *R*_S,*α*_ is a source resistance, *q*_out,*α*_ is the chamber outflow, *E*_nat,*α*_ and *E*_sep,*α*_ are the native chamber elastance and the septal elastance, respectively. Moreover, *p*_CL,*α*_ is the pressure in the contralateral chamber, *e.g.*, *p*_CL,LA_ = *p*_RA_.

Pericardium pressure is modeled as in (Sun et al., 1997), i.e., as an exponential function of the pericardial cavity volume

$$p_{\text{pc}}=K_{\text{pc}}\exp\left(\frac{V_{\text{pc}}-V_{0,pc}}{\Phi_{\text{pc}}}\right),$$ (10)

where *V*_0,pc_ is a volume offset, *K*_pc_ and Φ_pc_ are empirically determined constants and *V*_pc_ is the pericardium volume, computed as

$$V_{\text{pc}}=V_{\text{mio}}+V_{\text{pcf}}+\underset{\alpha }{\sum }V_{\alpha },$$ (11)

where *V*_mio_ and *V*_pcf_ are the volumes of the myocardium and the pericardial fluid, respectively, assumed to be constant.

Native elastance is defined as

$$E_{\text{nat},\alpha }=\frac{E_{\text{fw},\alpha }E_{\text{sep},\alpha }}{E_{\text{fw},\alpha }+E_{\text{sep},\alpha }}-\mu_{\text{AV},\alpha }q_{\text{V},\alpha }.$$ (12)

Here, *q*_V,*α*_ is the ventricular flow, which, together with constant *μ*_AV,*α*_, accounts for changes in effective atrial elastance caused by the movement of the atrio-ventricular plane, resulting in an enhancement of atrial filling. Moreover, *E*_fw,*α*_ is the free-wall elastance, given by

$$E_{\text{fw},\alpha }=k_{\alpha }\left(\frac{g_{1,\alpha }}{1+g_{1,\alpha }}\right)\left(\frac{1}{1+g_{2,\alpha }}\right)+E_{\text{fw},\alpha }^{\text{min}},$$ (13)

with

$$g_{1,\alpha }={\left(\frac{t-t_{\text{onset},\alpha }}{\tau_{1,\alpha }}\right)}^{m_{1,\alpha }},$$ (14)

$$g_{2,\alpha }={\left(\frac{t-t_{\text{onset},\alpha }}{\tau_{2,\alpha }}\right)}^{m_{2,\alpha }},$$ (15)

where *τ*_1,*α*_/*τ*_2,*α*_ are the contraction/relaxation time offsets, *m*_1,*α*_/*m*_2,*α*_ are the contraction/relaxation rate constants, *t*_onset,*α*_ is the contraction/relaxation time onset and *k*_*α*_ is

$$k_{\alpha }=\frac{E_{\text{fw},\alpha }^{\text{max}}-E_{\text{fw},\alpha }^{\text{min}}}{\underset{t\in \left[0,T\right]}{\max}\left[\left(\frac{g_{1,\alpha }}{1+g_{1,\alpha }}\right)\left(\frac{1}{1+g_{2,\alpha }}\right)\right]},$$ (16)

with $E_{\text{fw},\alpha }^{\text{max}}$ and $E_{\text{fw},\alpha }^{\text{min}}$ being the parameters that represent the maximum and minimum values of *E*_fw,*α*_.

Septal elastance is defined for inter-atrial and inter-ventricular septa. Therefore, this quantity is characterized as

$$E_{\text{sep},\alpha }=K_{\text{C}}E_{\text{fw,L}}+K_{\text{C}}E_{\text{fw,R}},$$ (17)

where $K_{\text{C}}$ is the septal elastance constant for atria (if *α* = LA or *α* = RA) and ventricles (if *α* = LV or *α* = RV), whereas pairs (L, R) are related to atria (LA, RA) or ventricles (LV, RV). Note that *E*_sep,LA_ = *E*_sep,RA_ and *E*_sep,LV_ = *E*_sep,RV_.

The source resistance *R*_S,*α*_ [see (Eq. 9)] is given by

$$R_{\text{S},\alpha }=K_{\text{S},\alpha }E_{\text{nat},\alpha }\left(V_{\alpha }-V_{0,\alpha }\right),$$ (18)

where *K*_S,*α*_ is a constant.

Cardiac valves are also modelled according to (Mynard, 2011; Mynard et al., 2012). Time rate of change for flow is given by

$${\dot{q}}_{\beta }=\frac{1}{L_{\beta }}\left(p_{\text{up},\beta }-p_{\text{down},\beta }-B_{\beta }q_{\beta }|q_{\beta }|\right),$$ (19)

with *β* ∈ {tv, pv, mv, av}, for tricuspid, pulmonary, mitral and aortic valves, respectively. Note that from now onwards we will use $\dot{v}=\frac{dv}{dt}$ for any time-dependent function *v*. Moreover, *p*_up,tv_ = *p*_RA_, *p*_up,pv_ = *p*_RV_, *p*_up,mv_ = *p*_LA_ and *p*_up,av_ = *p*_LV_, whereas *p*_down,tv_ = *p*_RV_, *p*_down,pv_ = *p*_pua_, *p*_down,mv_ = *p*_LV_ and *p*_down,av_ = *p*_ao_. Here, *p*_pua_ is the pressure of pulmonary arteries, to be introduced in Section 2.2.3, and *p*_ao_ is the pressure at the root of the aorta. Other parameters in (Eq. 19) are inertance *L*_*β*_ and resistance *B*_*β*_, which are computed as

$$L_{\beta }=\rho \frac{l_{\text{eff},\beta }}{A_{\text{eff},\beta }},\;$$ (20)

$$B_{\beta }=\frac{\rho }{2\;A_{\text{eff},\beta }^{2}}.$$ (21)

Where *l*_eff,*β*_ is a known effective valve length, whereas *A*_eff,*β*_ is the effective valve orifice area

$$A_{\text{eff},\beta }=\left(A_{\text{eff},\beta }^{\text{max}}-A_{\text{eff},\beta }^{\text{min}}\right)\zeta_{\beta }+A_{\text{eff},\beta }^{\text{min}},$$ (22)

which depends on valve state *ζ*_*β*_, the maximum effective orifice area $A_{\text{eff},\beta }^{\text{max}}$ and the minimum effective orifice area $A_{\text{eff},\beta }^{\text{min}}$ . In turn, valve state *ζ*_*β*_ is taken as

ζ˙β=Kvo,β1−ζβΔpβ,if Δpβ≥Δpopen,β,Kvc,βζβΔpβ,if ΔpβΔ<pclose,β,0,otherwise. (23)

with *K*_vo,*β*_ and *K*_vc,*β*_ being valve opening and closing constants, respectively. Moreover, Δ*p*_*β*_ = *p*_up,*β*_ − *p*_down,*β*_, while Δ*p*_open,*β*_ and Δ*p*_close,*β*_ are threshold opening and closing pressure differences.

#### 2.2.3 Pulmonary system

The pulmonary system is described by the model presented in (Sun et al., 1997). This model consists of three compartments: arteries (pua), capillaries (puc) and veins (puv). Each compartment is described as a CLR lumped parameter model. Compartment pressure is modeled as an exponential function of its volume

$$p_{\gamma }=E_{0,\gamma }V_{0,\gamma }\exp\left(\frac{V_{\gamma }}{V_{0,\gamma }}\right)+\Omega_{\gamma }\dot{V_{\gamma }},$$ (24)

with *γ* ∈ {pua, puc, puv}, where *V*_0,*γ*_ is a reference volume, related to a reference-volume elastance *E*_0,*γ*_ and Ω_*γ*_ is the viscoelastance of the compartment. Time evolution of volume is described by

$${\dot{V}}_{\gamma }=q_{\text{in},\gamma }-q_{\gamma },$$ (25)

where *q*_in,*γ*_ is the inlet flow for each compartment. In particular, we have that *q*_in,pua_ = *q*_pv_, *q*_in,puc_ = *q*_pua_ and *q*_in,puv_ = *q*_puc_. Moreover, flow time rate of change in each compartment is given by

$${\dot{q}}_{\gamma }=\frac{1}{L_{\gamma }}\left(p_{\gamma }-p_{\text{down},\gamma }-R_{\gamma }q_{\gamma }\right),$$ (26)

with *p*_down,pua_ = *p*_puc_, *p*_down,puc_ = *p*_puv_ and *p*_down,puv_ = *p*_LA_.

#### 2.2.4 Peripheral beds

In the current model there is no one-to-one relation between arterial and venous terminals of one-dimensional networks. Connectivity between terminal arteries and their venous counterpart was defined in Section 2.1.3.

Peripheral circulation was divided into two compartments: a proximal compartment (corresponding to arteriolar/capillary circulation) and a distal compartment (corresponding to venules/small veins). As a result of these modeling choices, each terminal artery gives rise to a proximal compartment, which was in turn linked to $N_{\text{ven}}^{\theta }$ distal compartments. On the other hand, each terminal vein gives rise to a distal compartment, which was linked to $N_{\text{art}}^{\theta }$ proximal compartments. This situation is illustrated in Figure 3, with a specific example. The proximal compartment was linked to the distal compartment via a proximal resistance *R*_*a*_. Then, proximal and distal compartments where linked by a resistance *R*_*a*,*v*_, while the distal compartment was linked to the corresponding terminal vein via a distal resistance *R*_*v*_.

Peripheral circulation is modeled as a series of elastic compartments that can accumulate blood and dissipate energy due to friction by using standard RCR lumped parameter models. In such models pressure is a linear function of volume

$$p_{\theta }=\frac{V_{\theta }}{C_{\theta }}+p_{\text{ext},\theta },$$ (27)

where *C*_*θ*_ is the compliance of compartment *θ*, *p*_ext,*θ*_ is the external pressure acting on this compartment and *V*_*θ*_ is the compartment volume, whose time rate of change is defined by the mass conservation principle

$${\dot{V}}_{\theta }=q_{\text{in},\theta }-q_{\text{out},\theta }.$$ (28)

For proximal compartments we have that $q_{\text{in},a_{i}}$ is computed by imposing mass and energy conservation at the interface between the one-dimensional terminal artery outlet and the lumped-parameter model. The same is valid for $q_{\text{out},v_{j}}$ , which in this case is computed by imposing coupling conditions that guarantee mass conservation and momentum balance at the interface between the terminal vein inlet and the lumped parameter model. For details see (Müller and Toro, 2014b; Toro et al., 2022). Flow leaving proximal arterial compartments is computed as

$$q_{\text{out},a_{i}}=\overset{N_{\text{ven},i}}{\underset{l=1}{\sum }}\frac{p_{a_{i}}-p_{v_{l}}}{R_{a_{i},v_{l}}}.$$ (29)

In turn, flow entering a venous compartment is computed as

$$q_{\text{in},v_{j}}=\overset{N_{\text{art},j}}{\underset{l=1}{\sum }}\frac{p_{a_{l}}-p_{v_{j}}}{R_{a_{l},v_{j}}}.$$ (30)

#### 2.2.5 Coronary beds

While the approach for defining arterio-venous connections of coronary peripheral beds is identical to the one previously exposed, the mathematical model used here differs in order to account for the specificity of blood flow dynamics in the cardiac microcirculation. In fact, here we replace the model defined by Eqs 27, 28 with the model proposed by (Mynard et al., 2014). If we consider the connection between a proximal and a distal compartment, instead of having a simple resistance (*R*_*a*,*v*_) connecting both models we have the circuit depicted in (Mynard et al., 2014, Figure 2). In practice, the peripheral vascular bed is divided into three layers, corresponding to the sub-epicardium, the midwall and the sub-endocardium. Each layer is further divided into two regions, each having a corresponding compliance and an associated volume. Moreover, each layer contains three varying resistances, whose values are a function of the volume of the compartment’s region, and are subjected to the intramyocardial pressure. According to (Mynard et al., 2014), the volumes for each layer of a given compartment are defined as

$$V_{i,\lambda }=V_{i,\lambda }^{0}+C_{i,\lambda }p_{i,\lambda }^{\text{tm}},$$ (31)

with *i* = 1, 2 and *λ* ∈ {sub-epi, midwall, sub-endo}. We note here that we have dropped the index that identifies the specific compartment for the sake of clarity. However, it must be noted that the equations presented in this section are valid for each coronary arterial-venous connection. Here, $V_{i,\lambda }^{0}$ is a reference volume and *C*_*i*,*λ*_ is the compliance for *i*th compartment region. Moreover, $p_{i,\lambda }^{\text{tm}}$ is the *λ*-layer transmural pressure for region *i*, defined as

$$p_{i,\lambda }^{\text{tm}}=p_{i,\lambda }-p_{\text{im},\lambda },$$ (32)

where *p*_*i*,*λ*_ is the blood pressure and *p*_im,*λ*_ is the intramyocardial pressure, defined as

$$p_{\text{im},\lambda }={CEP}_{\lambda ,\phi }+{SIP}_{\phi },$$ (33)

CEP_*λ*,*ϕ*_ stands for cavity-induced extracellular pressure and is considered to vary linearly between the chamber pressure and the pericardium pressure, while SIP_*ϕ*_ is the shortening-induced intracellular pressure. In the current model, coronary vessels perfuse the free walls of the four heart chambers, as well as the interventricular septa (IVS). Therefore, CEP and SIP are defined for each one of these portions of the myocardium. Following (Mynard et al., 2014), we consider CEP as

$${CEP}_{\lambda ,\phi }=p_{\text{out},\phi }+w_{\lambda }\left(p_{\text{ch},\phi }-p_{\text{out},\phi }\right),$$ (34)

with *ϕ* ∈ {RA-fw, RV-fw, LA-fw, LV-fw, IVS}. Also, it is *w*_sub-endo_ = 5/6, *w*_midwall_ = 1/2 and *w*_sub-epi_ = 1/6. For free walls we set *p*_out,RA-fw_ = *p*_out,RV-fw_ = *p*_out,LA-fw_ = *p*_out,LV-fw_ = *p*_pc_, while for inter-ventricular septa we take *p*_out,IVS_ = *p*_RV_. Moreover, *p*_ch,*ϕ*_ is the chamber pressure whose wall is perfused by the compartment. For IVS we set *p*_ch,*ϕ*_ = *p*_LV_, whereas for free walls the choice is obvious. Finally, SIP is defined as

$${SIP}_{\phi }=\alpha_{\text{SIP}}\left(\frac{p_{\text{ch},\phi }}{V_{\text{ch},\phi }-V_{\text{ch},\phi }^{0}}\right),$$ (35)

where *α*_SIP_ = 8.2, while chamber ch is chosen as for the computation of CEP_*λ*,*ϕ*_.

Each coronary vascular territory has 9 resistances, i.e., $R_{\lambda }^{i}$ , with *i* ∈ {1, m, 2} and *λ* ∈ {sub-epi, midwall, sub-endo}. The relation between resistance and volume, proposed in (Mynard et al., 2014), is as follows

$$R_{\lambda }^{j}=R_{\lambda ,0}^{j}{\left(\frac{V_{\lambda ,0}^{j}}{V_{\lambda }^{j}}\right)}^{2},$$ (36)

with *j* ∈ {1, 2} and

$$R_{\lambda }^{m}=R_{\lambda ,0}^{m}\left[\frac{3}{4}{\left(\frac{V_{\lambda ,0}^{1}}{V_{\lambda }^{1}}\right)}^{2}+\frac{1}{4}{\left(\frac{V_{\lambda ,0}^{2}}{V_{\lambda }^{2}}\right)}^{2}\right],$$ (37)

where $R_{\lambda ,0}^{j}$ and $R_{\lambda ,0}^{m}$ are nominal resistances for $V_{\lambda }^{j}=V_{\lambda ,0}^{j}$ , and $V_{\lambda ,0}^{j}$ are compartment volumes for zero transmural pressure.

#### 2.2.6 Intracranial pressure

The interaction between the cerebral vasculature and intracranial pressure (ICP) is taken into account by adopting the model proposed by (Ursino, 1988; Ursino and Lodi, 1997). It is worth noting that this model was first coupled to a one-dimensional model of the arterial and venous circulation in (Müller and Toro, 2014a). The time rate of change of intracranial pressure is given by

$${\dot{p}}_{\text{ICP}}=\frac{1}{C_{\text{ICP}}}\left({\dot{V}}_{\text{cbv}}+q_{\text{gen}}-q_{\text{abs}}\right),$$ (38)

where *V*_cbv_ is the cerebral blood volume, *q*_gen_ and *q*_abs_ are cerebrospinal fluid generation and absorption rates and *C*_ICP_ is the cranio-spinal cavity compliance, given by

$$C_{\text{ICP}}=\frac{1}{k_{\text{ICP}}p_{\text{ICP}}},$$ (39)

where *k*_ICP_ is an experimentally determined coefficient. Since in this work we do not assess pathological perturbations to the baseline state, we will consider *q*_gen_ = *q*_abs_.

#### 2.2.7 Venous valves

Venous valves are modeled as described in (Mynard et al., 2012). The lumped parameter model proposed there is identical to the one described by Eqs 19–23. The only difference with respect to cardiac valves is that here the lumped parameter model representing the valve is placed between two one-dimensional segments representing a vein.

#### 2.2.8 Starling resistors

As originally proposed in (Müller and Toro, 2014a), it is important to include particular non-linear resistances close to the point where intracranial veins meet dural sinuses. These non-linear resistances are called Starling resistors and provide a mechanism through which the downstream haemodynamic conditions are decoupled from the haemodynamic state at intracranial veins, whenever dural sinus pressures are lower than the intracranial pressure. Here, instead of using the ideal diode model proposed in (Müller and Toro, 2014a), we propose an alternative model, built on the valve model proposed by (Mynard, 2011). Starling resistors (SR) are lumped parameter models placed between two one-dimensional segments. They are located at the point where intracranial veins join dural sinuses (see Figure 2). Here, the time rate of change of flow is given by

$${\dot{q}}_{\omega }=\frac{1}{L_{\omega }}\left(p_{\text{up},\omega }-p_{\text{down},\omega }^{*}-B_{\omega }q_{\omega }|q_{\omega }|\right),$$ (40)

where *B*_*ω*_ and *L*_*ω*_ are computed as indicated for valves in Section 2.2.2. While *p*_up,*ω*_ is the pressure in the upstream vessel, the downstream pressure is computed as

$$p_{\text{down},\omega }^{*}=\left(p_{\text{down},\omega }-p_{\text{ext},\omega }\right)\zeta_{\omega }+p_{\text{ext},\omega },$$ (41)

which depends on the SR state *ζ*_*ω*_. In this way we have that for an open SR the downstream blood pressure is the pressure used to compute flow accross the SR, while in the case of a closed/collapsed SR the driving pressure is the external one, which, in turn, for cerebral vessels corresponds to the intracranial pressure. The time rate of change of *ζ*_*ω*_ is defined as

$$\dot{\zeta_{\omega }}=\left\{\begin{array}{cccc} & K_{\text{so},\omega }\left(1-\zeta_{\omega }\right)\Delta p_{\omega }, & \text{if } & \Delta p_{\omega }\geq 0,\\ & K_{\text{sc},\omega }\zeta_{\omega }\Delta p_{\omega }, & \text{if } & \Delta p_{\omega }<0, \end{array}\right.$$ (42)

with *K*_so,*ω*_ and *K*_sc,*ω*_ being SR opening and closing constants, respectively. Moreover, Δ*p*_*ω*_ = *p*_down,*ω*_ − *p*_ext,*ω*_.

#### 2.2.9 Coupling conditions

Coupling conditions reported in (Eqs 7, 8) are not the only wave relations used to couple one-dimensional domains at bifurcation/junction points. In fact other coupling conditions are needed. These regard generalized Riemann invariants, which are quantities preserved along characteristics for hyperbolic systems of partial differential equations. These quantities are also used to couple one-dimensional domains to peripheral circulation models (for terminal arteries and veins), to the right atrium (for inferior and superior caval veins), and to the aortic valve (for the ascending aorta). In all these cases generalized Riemann invariants as well as additional conditions enforcing mass and energy conservation at discrete level are used to compute coupling conditions. These aspects are explained in full detail in (Müller and Toro, 2014b) and (Müller et al., 2016a).

### 2.3 Numerical methods

The methodology for the discretization of the one-dimensional blood flow model, as well as its coupling to lumped parameter models, was presented in several previous works by the authors. Here we provide a brief description of the main aspects of the numerical methods used to discretize the underlying partial and ordinary differential equations, providing relevant references for each aspect.

The one-dimensional blood flow model defined by Eqs 1, 2, which constitutes an advection-diffusion-reaction system, is hyperbolized following the approach presented in (Montecinos and Toro, 2014; Toro and Montecinos, 2014) in order to obtain a system of first order partial differential equations. The hyperbolic character of the resulting first order system holds under certain assumptions, which include parameter and state ranges encountered in biomedical applications. For further details on the resulting hyperbolized system refer to (Montecinos et al., 2014; Müller et al., 2016a; Müller et al., 2016b).

The hyperbolized system is discretized with an explicit, local time stepping, second order finite volume scheme (Müller et al., 2016a), which ensures the preservation of high-order accuracy at junctions (Müller and Blanco, 2015). The numerical method is based on the ADER high-order numerical framework, first reported in (Toro et al., 2001), and for which an up-to-date review is provided in (Toro, 2020). Here we use the Dumbser-Enaux-Toro method to solve the generalized Riemann problem (Dumbser et al., 2008), since this solver can deal with the stiff source term emerging from the hyperbolic reformulation in a robust manner. The employed numerical scheme belongs to the family of path-conservative schemes (Parés, 2006), while for the computation of numerical fluctuations we used the modification of the Dumbser-Osher-Toro solver (Dumbser and Toro, 2011a; Dumbser and Toro, 2011b) proposed in (Müller et al., 2016b), which is well-balanced for varying mechanical and geometrical properties along vessels. Furthermore, the scheme applies consistent coupling conditions at junctions of viscoelastic vessels (Müller et al., 2016b). The local time stepping technique employed here was proposed in (Müller et al., 2016a), as an adaptation of the method presented in (Dumbser et al., 2007) to networks of one-dimensional domains. The maximum local time step is set to Δ*t*_max_ = 1 *ms*, whereas the time step in each vessel is computed in such a way that ensures synchronization of time at all junctions of the network and non-violation of the Courant-Friedrichs-Lewy stability condition, for which *CFL* = 0.9 is used. Since wave speeds for the hyperbolized system are much bigger than blood velocities, we consider constant time steps along the simulation, but in principle the local time step could be adapted to local flow conditions in run-time. As for the spatial discretization, the characteristic mesh spacing is Δ*x*_*c*_ = 1 *cm*, vessels shorter than Δ*x*_*c*_ are discretized with a single computational cell.

Ordinary differential equations for lumped parameter models are discretized with an explicit Euler method. Its coupling to one-dimensional vessels is described in (63). Lumped parameter models for valves and SRs have a time step equal to the local time step of the vessels where they are located. From this observation it follows that the time step of two vessels connected by a valve or a SR are forced to be equal. The time step for lumped parameter models regarding the heart and the pulmonary circulation are equal and are determined in the same way as done for the time integration along junctions, see (Müller et al., 2016a) for details. The time step for the discretization of Eq. 38, regarding the ICP, is taken equal to Δ*t*_max_.

Simulations shown in this work were performed using eight parallel processes with 8 threads each, yielding a wall-clock simulation time for a cardiac cycle of approximately 15 min on computational nodes equipped with Intel^®^ Xeon^®^ CPU E5-2650 v2 @ 2.60 GHz processors.

### 2.4 Model parameters

Here we describe the setting of model parameters for all the components of the model, maken also reference to the provided suplementary material.

#### 2.4.1 Blood behavior

Blood is considered as a Newtonian fluid with viscosity *μ* = 0.04 *P* everywhere expect in perforator arteries, for which *μ*_per_ = 0.01 *P* is used. For a discussion on this modelling choice refer to (Blanco et al., 2014). Blood density is *ρ* = 1.04 *g*/*cm* ^3^ for all vascular districts included in the model. A fully developed parabolic velocity profile is considered, resulting in the friction coefficient *f* previously specified.

#### 2.4.2 Wall behavior

As previously stated, different tube laws are used for arteries and veins. Here we describe how parameters found in tube laws (Eqs 3, 6) are determined for all vessels of the ADAVN model.

##### 2.4.2.1 Arteries

Effective Young moduli *E*_*e*_ and *E*_*c*_ and viscoelastic coefficient $K_{m}^{\text{art}}$ present different values according to the vessel size. In particular, vessels are divided in three categories according to their radii as shown in Table 1. Then, effective coefficients for the different categories are computed by using a simple mixture theory approach, namely, we have $E_{e}^{j}=W_{e}^{j}\;E_{e}$ , $E_{c}^{j}=W_{c}^{j}\;E_{c}$ and $K_{m}^{j}=W_{m}^{j}\;K_{m}^{\text{art}}$ with *j* = {*A*, *B*, *C*} and *E*_*e*_ = 4.0 × 10^6^ *dyn*/*cm* ^2^, *E*_*c*_ = 1.0 × 10^9^ *dyn*/*cm* ^2^ and $K_{m}^{\text{art}}=3.6\times 10^{5}\;dyn\;s/cm^{2}$ . Weights for the mixture theory approach are provided in Table 2.

**TABLE 1:** Arterial vessel groups according to lumen radius.

Group	Lumen radius [*cm*]
*A*	*R*_0_ > 0.18
*B*	0.07 ≤ *R*_0_ ≤ 0.18
*C*	0.07 > *R*_0_

**TABLE 2:** Arterial vessel wall constituent fractions.

Group	*A*	*B*	*C*
*W*_*E*_	0.85	0.65	0.45
*W*_*C*_	0.05	0.20	0.00
*W*_*M*_	0.10	0.15	0.55

Wall thickness for arteries is computed according to the vessel radius by

$$\frac{h_{0}}{R_{0}}=a\exp^{bR_{0}}+c\exp^{dR_{0}},$$ (43)

with *a* = 0.2802, *b* = −5.053 *cm* ^−1^, *c* = 0.1324 and *d* = −0.1114 *cm* ^−1^. The pressure of the reference state is $p_{0}^{\text{art}}=10^{5}\;dyn/cm^{2}$ . Finally, the deformation state for collagen fiber activation is set to *ɛ*_0_ = 0.25 and the standard deviation of the fiber activation state distribution is taken as *ϵ*_*r*_ = 0.05.

##### 2.4.2.2 Veins

In this case we follow the approach proposed in (Müller and Toro, 2014b). For dural sinuses we use *m* = 1/2 and *n* = 0, while for all other veins we set *m* = 10 and *n* = −3/2. The pressure of the reference state is $p_{0}^{\text{ven}}=6666.66\;dyn/cm^{2}$ . Moreover, the stiffness coefficient *K* is computed by assuming that celerity *c* is related to the venous radius

$$c_{\text{ven}}=c_{\text{ven}}^{\text{max}}-\left(c_{\text{ven}}^{\text{max}}-c_{\text{ven}}^{\text{min}}\right){\left(\frac{\hat{R}-R_{\text{ven}}^{\text{min}}}{R_{\text{ven}}^{\text{max}}-R_{\text{ven}}^{\text{min}}}\right)}^{\frac{1}{2}},$$ (44)

with $\hat{R}=\max\left(R_{\text{ven}}^{\text{min}},R_{0}\left(x_{\text{mid}}\right)\right)$ . Here *x*_mid_ is the vessel’s midpoint coordinate, while $R_{\text{ven}}^{\text{min}}=0.08\;cm$ and $R_{\text{ven}}^{\text{max}}=0.80\;cm$ . Once that *c*_ven_ is known, the stiffness coefficient is computed from the celerity function evaluated at *A*_0_, i.e.,

$$K=\frac{\rho c_{\text{ven}}^{2}}{m-n}.$$ (45)

Finally, the viscoelastic coefficient in this case is computed as in (Mynard and Smolich, 2015), using a known relation between the vessel radius and this coefficient. Here we set $K_{m}^{\text{ven}}=\hat{K}R_{0}$ , with $\hat{K}=708.98\;dyn/cm^{2}\;s$ . This value is based on considerations of the vessel wall thickness-to-radius ratio, the percentage of smooth muscle cells in veins and the relation between material viscosity and coefficient *K*_*m*_ (Alastruey et al., 2011). As it will be seen later on, this choice guarantees physiologically-sound pressure-area loops.

#### 2.4.3 Cardiac and pulmonary parameters

Parameters for the heart model are provided in Tables S1, S2 and S3 in Supplementary Appendix A1, for cardiac chambers, pericardium and cardiac valve models, respectively. Such parameters are based on values reported in (Mynard and Smolich, 2015) with slight modifications. Parameters for the pulmonary circulation model are reported in Table S4 in Supplementary Appendix A1 and are based on values reported in (Sun et al., 1997).

#### 2.4.4 1D network characteristics and stretched volume

The Supplementary File adavn_vessels.csv provides information about the connectivity of all vessels of the model, shown in Figure 1, their length, initial and final radii, as well as the body region to which the vessel belongs, according to region numbers provided in Table S6 in Supplementary Appendix A1. The total stretched volume is set to $V_{\text{stretched}}^{\text{set}}=2273.643\;cm^{3}$ and it is enforced by computing the stretched volume $V_{\text{stretched}}^{0}$ at the beginning of a simulation and injecting/extracting the difference $V_{\text{stretched}}^{\text{set}}-V_{\text{stretched}}^{0}$ during the first 3 s of the simulation.

#### 2.4.5 General peripheral beds

In this work we preserved the blood flow distribution of the ADAN model, as defined in (Blanco et al., 2014). Each terminal artery in ADAN model is equipped with a total peripheral resistance $R_{t}^{k}$ and a total residual arterial compliance $C_{a}^{k}$ .

We recall that in our generic peripheral circulation model *θ* there are $N_{\text{art}}^{\theta }$ contributing arteries and $N_{\text{ven}}^{\theta }$ draining veins. Then, a generic *k*th terminal artery is linked to an *i*th proximal elastic compartment with compliance $C_{ai}=C_{a}^{k}$ , connected to the terminal artery by a proximal resistance $R_{ai}=0.15\;R_{t}^{k}$ . In turn, the *i*th proximal elastic compartment can be connected to *N*_ven,*i*_ veins by resistances $R_{a_{i},v_{l}}$ , with *l* = 1, … , *N*_ven,*i*_ and equivalent resistance $R_{a_{i},equiv}=0.85\;R_{t}^{k}$ . This resistance is in turn distributed among the $R_{a_{i},v_{l}}$ resistances proportionally to the cubed radii of the *N*_ven,*i*_ veins to which the *i*th proximal elastic compartment is connected.

The *j*th terminal vein represents an elastic venous compartment, connected to the terminal vein via a proximal resistance *R*_*vj*_, computed as the characteristic impedance of the terminal vein $R_{vj}=\rho *c\left(\hat{A}\right)/\hat{A}$ , with $\hat{A}$ the area of the vein at its terminal point. Finally, the compliance of the venous elastic compartment is computed as follows. First we define a total systemic venous compliance *C*_*v*,data_ = 146 *mL*/*mmHg*, from which we compute a residual compliance *C*_*v*,res_ by subtracting the compliance of all one-dimensional venous domains *C*_*v*,1*D*_. This residual compliance is distributed among all venous elastic compartments by considering the ratio between the compliance of contributing proximal elastic compartments *C*_*ai*_ over total arterial peripheral compliance *C*_tot_, since in (Blanco et al., 2014) *C*_*ai*_ was determined according to blood flow distribution, so that the ratio *C*_*ai*_/*C*_tot_ is proportional to flow distribution.

The total number of peripheral circulation units is 60. The Supplementary File adavn_vessels.ods includes, for terminal arteries, total peripheral resistance $R_{t}^{k}$ and a total residual arterial compliance $C_{a}^{k}$ , as well as arterio-venous connectivity, and the vascular territory code to which terminal vessels belong.

#### 2.4.6 Coronary beds

$V_{\lambda ,0}^{i}$ , with *i* ∈ {1, 2} and *λ* ∈ {sub-epi, midwall, sub-endo}, are compartment volumes for zero transmural pressure and are determined according to $V_{\text{perf}}^{i}$ , the volume of myocardial tissue perfused by each terminal coronary artery. $V_{\text{perf}}^{i}$ is determined by subdividing a total myocardial volume of 246.38 *cm* ^3^, into left ventricle free wall volume (111.43 cm^3^), right ventricle free wall volume (47.62 cm^3^), interventricular septum volume (58.09 cm^3^), left atrial wall volume (15.48 cm^3^) and right atrial wall volume (13.76 cm^3^), as specified in (Lorenz et al., 1999), identifying the terminal coronary arteries perfusing each of these structures and assuming that blood flow is proportional to the cube of the terminal radius or arteries. The computed perfused myocardial tissue volume $V_{\text{perf}}^{i}$ for each terminal artery is provided in Table S5 in Supplementary Appendix A1. This volume is then further subdivided depending on whether the artery connects to one or two venous compartments, as done for total peripheral arterial resistance $R_{a_{i},v_{l}}$ in Section 2.4.5. Once the perfused mycardium volume is available for each arterio-venous connection, the actual blood volumes are computed assuming that the perfusion rates are different, i.e.,

$$V_{\text{tot},0}^{i}=V_{\text{perf}}^{i}\;\rho_{\text{myo}}\;\frac{\eta_{i}}{100g},$$ (46)

with *η*_1_ = 2.5 *cm* ^3^/100*g*, *η*_2_ = 8.0 cm^3^/100*g* and *ρ*_myo_ = 1.05 *g*/*cm* ^3^ the density of myocardial tissue. Then, each $V_{\text{tot},0}^{i}$ is further subdivided according to the following relation

$$V_{\text{tot},0}^{i}=V_{\text{sub}-\text{endo},0}^{i}+V_{\text{midwall},0}^{i}+V_{\text{sub}-\text{epi},0}^{i},$$ (47)

assuming that $V_{\text{midwall},0}^{i}=0.93\;V_{\text{sub}-\text{endo},0}^{i}$ and that $V_{\text{sub}-\text{epi},0}^{i}=0.87\;V_{\text{sub}-\text{endo},0}^{i}$ .

The same approach is followed for compliances $C_{\lambda }^{i}$ , but in this case we have that

$$C_{\text{tot}}^{i}=V_{\text{perf}}^{i}\;\rho_{\text{myo}}\;\frac{\kappa_{i}}{100g},$$ (48)

with *κ*_1_ = 9.7501 10^−6^ *cm* ^5^/*dyn*/100*g* and *κ*_2_ = 1.905019 10^−4^ *cm* ^5^/*dyn*/100*g* (Mynard et al., 2014), while the subdivision among the three layers is performed using the same criteria used for volumes.

The compliance of arterioles, i.e., the capacitor directly connected to a terminal coronary artery, is computed as $0.1\;\sum_{k}C_{\text{tot}}^{1,k}$ , with *k* = 1, … , *N*_a_, where *N*_a_ is the number of arterio-venous connections departing from a terminal coronary artery. In turn, the compliance of the capacitor attached to a terminal vein is computed as $0.1\;\sum_{k}C_{\text{tot}}^{2,l}$ , with *l* = 1, … , *N*_v_, where *N*_v_ is the number of arterio-venous connections draining into a terminal coronary vein.

In order to define $R_{\lambda ,0}^{i}$ we assume that the three vascular layers are connected in parallel, so that we can write

$$\frac{1}{R_{\text{tot},0}}=\frac{1}{R_{\text{midwall},0}^{\text{tot}}}+\frac{1}{R_{\text{sub}-\text{endo},0}^{\text{tot}}}+\frac{1}{R_{\text{sub}-\text{epi},0}^{\text{tot}}},$$ (49)

and assume that $R_{\text{midwall},0}^{\text{tot}}=0.6\;R_{\text{sub}-\text{epi},0}^{\text{tot}}$ and $R_{\text{sub}-\text{endo},0}^{\text{tot}}=0.2\;R_{\text{sub}-\text{epi},0}^{\text{tot}}$ . Next, $R_{\lambda ,0}^{\text{tot}}$ is further subdivided into $R_{\lambda ,0}^{i}$ , with *i* ∈ {1, m, 2} by noting that they are connected in series

$$R_{\lambda ,0}^{\text{tot}}=\underset{i\in \left\{1,\text{m},2\right\}}{\sum }R_{\lambda ,0}^{i},$$ (50)

and assuming that $R_{\lambda ,0}^{1}=1.2R_{\lambda ,0}^{m}$ and $R_{\lambda ,0}^{2}=0.5R_{\lambda ,0}^{m}$ . *R*_tot,0_ is the total peripheral arterial resistance for each arterio-venous connection $R_{a_{i},v_{l}}$ , introduced in Section 2.4.5.

All parameters values used for coronary peripheral beds are based on parameters proposed in (Mynard and Smolich, 2015), with resistances slightly modified to obtain a total coronary flow equal to approximately 4.5% of the cardiac output.

#### 2.4.7 Intracranial pressure model

The only parameter involved in the intracranial pressure model is the proportionality constant for the nonlinear cranio-spinal cavity compliance. According to previous work, this parameter is set to *k*_ICP_ = 0.15 *mL* ^−1^. We used *p*_ICP_ = 14665.42 *dyn*/*cm* ^2^ as initial condition, along with Eq. 38, to describe intracranial pressure time evolution.

#### 2.4.8 Valves and Starling resistors

Tables S7 and S8 in Supplementary Appendix A1 provide information on the location of valves and Starling resistors. The maximum effective orifice area $A_{\text{eff}}^{\text{max}}$ and the effective lengths *l*_eff_ were computed as the average of the reference area and diameter of proximal and distal venous segments connected to the valve/Starling resistor. Finally, for all venous valves we used *K*_vo_ = 0.1, *K*_vc_ = 0.03, while for all Starling resistors we set *K*_so_ = 0.01, *K*_sc_ = 0.01.

### 2.5 Local sensitivity analysis

We computed local sensitivity indexes defined by

$$S_{M,P}^{\pm }=\frac{{\hat{M}}^{\pm }-M}{M}\;100,$$

where M is the baseline value of a variable for which we want to compute local sensitivity indexes and ${\hat{M}}^{\pm }$ is the value of the variable of interest obtained by increasing/decreasing (+/−) parameter P by 10% of its reference value.

## 3 Results

In this section we illustrate the performance of ADAVN concerning its capacity to decribe haemodynamics in terms of quantitative indexes as well as pressure, velocity and flow rate waveforms. Local sensitivity analysis results are also included.

### 3.1 Haemodynamic variables

Table 3 reports the values of typical cardiovascular indexes computed with the ADAVN model. Also, reference values extracted from the literature have been included. Such indexes regard cardiac and vascular performance, as well as flow distribution in the two vascular territories focused by the venous system description, namely, cerebral and coronary veins. The definition of these indexes is provided in Supplementary Appendix B1.

**TABLE 3:** Main model-predicted cardiac and haemodynamic variables and reference values reported in the literature.

Heart and ventriculo-arterial coupling				
Parameter	Units	Model	Ref. val	Ref
LVSV	mL	79.02	(40–120)	Levick (2010)
LVEF	%	64.04	(52–72)	Kosaraju et al. (2022)
E_LV_I	mmHg/mL * m^2^	3.91	4.50(−)	Najjar et al. (2004)
EaI	mmHg/mL * m^2^	2.05	2.20(−)	Najjar et al. (2004)
EaI/E_LV_I	-	0.56	0.58(−)	Najjar et al. (2004)
Pressures				
Parameter	Units	Model	Ref. val	Ref
MAP	mmHg	92.58	88(8)	McEniery et al. (2005)
DBP	mmHg	71.82	73(8)	McEniery et al. (2005)
SBP	mmHg	110.1	123(10)	McEniery et al. (2005)
MPAP	mmHg	14.98	14(3)	Lau et al. (2016)
ICP	mmHg	11.02	(5–15)	Rangel-Castillo et al. (2008)
CVP	mmHg	4.68	(0–5)	Levick (2010)
PWV_CF_	cm/s	495.23	(550–1,100)	Yu et al. (2008)
PWV_FP_	cm/s	728.61	(600–1,000)	Sugawara et al. (2009)
ABI	-	1.15	(1.11–1.40)	Fowkes et al. (2008)
PPA	-	1.14	(1.5.1.9)	Avolio et al. (2009)
PP_A_	mmHg	33.59	30(6)	McEniery et al. (2005)
PP_F_	mmHg	56.75	50(9)	McEniery et al. (2005)
Blood flow distribution				
Cardiac cycle average flow	Units	Model	Ref. val	Ref
Cardiac output	mL/s	96.36	83.3(33.3)	Cattermole et al. (2017)
Cerebral blood flow	mL/s	12.45	12.18(2.12)	Ford et al. (2005)
Coronary blood flow	mL/s	4.90	4.5(1.36)	Sakamoto et al. (2013)
Int. Carotid Art	mL/s	4.56	4.62(0.93)	Ford et al. (2005)
Vert. Artery	mL/s	1.24	1.32(0.72)	Ford et al. (2005)
Left Ant. Desc. Artery	mL/s	1.55	1.40(0.67)	Sakamoto et al. (2013)
Left circumflex Artery	mL/s	1.22	1.20(0.62)	Sakamoto et al. (2013)
Right Coronary Artery	mL/s	2.09	1.88(0.82)	Sakamoto et al. (2013)

LVSV: left ventricle stroke volume; LVEF: left ventricle ejection fraction; E_LV_I: left ventricle elastance index; EaI: arterial elastance index; MAP/SBP/DBP: mean/systolic/diastolic blood pressure; MPAP: mean pulmonary arterial pressure; ICP: intracranial pressure; CVP: central venous pressure; PWV_CF_: carotid-femoral pulse wave velocity; PWV_FP_: femoral-posterior tibial pulse wave velocity; ABI: ankle-brachial index; PPA: pulse pressure amplification; PP_A_: pulse pressure in the ascending aorta; PP_F_ pulse pressure in the left femoral artery. A precise description of the computation of reported indexes is provided in Supplementary Appendix B1.

### 3.2 Haemodynamic waveforms

**FIGURE 4:**
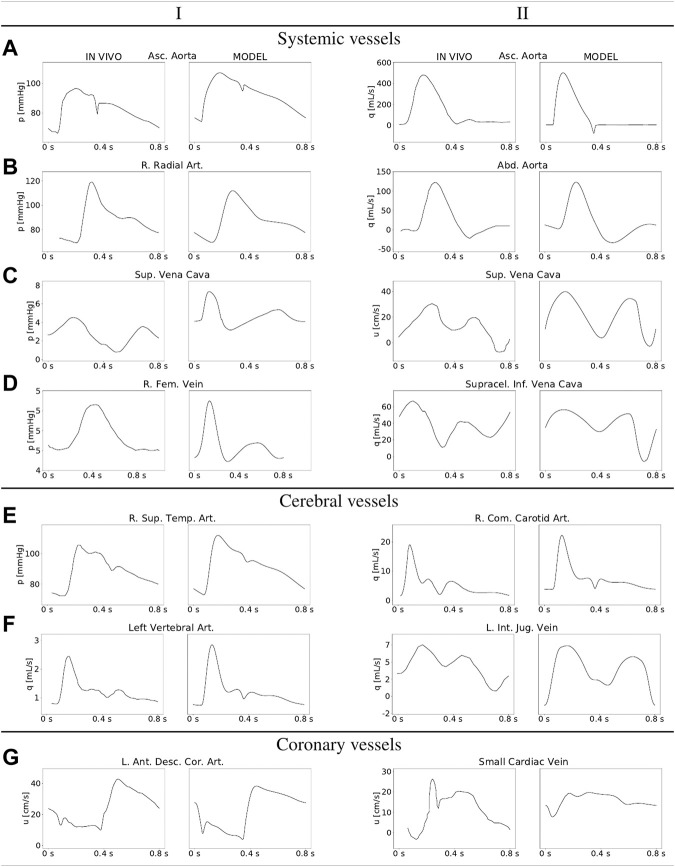
Comparison of *in vivo* and model-predicted haemodynamic waveforms for selected systemic, cerebral and coronary vessels. Full names, index according to the Supplementary File adavn_vessels.csv and the bibliographic reference from which the *in-vivo* data was digitalized: Asc. Aorta (press. and flow): Ascending Aorta, 816, (Murgo et al., 1980; Reymond et al., 2009); R. Radial Art.: Right Radial Artery, 3168, (Reymond et al., 2009); Abd. Aorta: Abdominal Aorta, 843, (Reymond et al., 2009); Sup. Vena Cava (press. and vel.): Superior Vena Cava, 4288, (Cohen et al., 1986); R. Fem. vein: Right Femoral Vein, 4165 (Chang et al., 2020),*; Supracel. Inf. Vena Cava: Supraceliac Inferior Vena Cava, 4148, (Cheng et al., 2002); R. Sup. Temp. Art.: Right Superior Temporal Artery, 212, (Reymond et al., 2009); R. Com. Carotid Art.: Right Common Carotid Artery, 736, (Gwilliam et al., 2009); L. Vertebral Art.: Left Vertebral Artery, 2504, (Reymond et al., 2009); L. Int. Jug. Vein: Left Internal Jugular Vein, 4058, (Müller and Toro, 2014a); L. Ant. Desc. Cor. Art.: Left Anterior Descending Coronary Artery, 1569, (Davies et al., 2006); Small Cardiac Vein: 4265, (Kajiya et al., 1993).

Figure 4 provides a comparison of model-predicted waveforms *versus in-vivo* signals for selected arteries and veins in the systemic, coronary and cerebral circulations.

### 3.3 Local sensitivity analysis

Variables considered in the computation of local sensitivity indexes are $M=\left\{MAP,{PP}_{\text{A}},CVP,CO\right\}$ , where:

•MAP is the mean arterial pressure, computed at the midpoint of the ascending aorta (vessel with index 816 according to the Supplementary File adavn_vessels.csv);
•PP_A_ is the aortic pulse pressure, i.e., the difference between maximum and minimum pressure values over a cardiac cycle for the ascending aorta;
•CVP is the cardiac cycle-averaged pressure in the right atrium;
•CO is the cardiac output, i.e., the cardiac cycle-averaged flow rate in the ascending aorta.

Local sensitivity was computed for all model parameters, excluding network geometry and topology. The total number of considered parameters was 96 and consequently the total number of performed simulations was 193, if the reference configuration is included. Table 4 shows the local sensitivity indexes for the main cardiovascular markers included in the set M .

**TABLE 4:** Local sensitivities $S_{M,P}^{\pm }$ (and percentage change with respect to nominal values shown in top row) of variables $M=\left\{MAP,{PP}_{\text{A}},CVP,CO\right\}$ . Sensitivities are ranked according to their absolute values. Only the first 20 most influential parameters are reported.

Rank	MAP (92.58 mmHg)	PP_A_ (33.08 mmHg)	CVP (4.67 mmHg)	CO (96.36 mL/s)
1	$V_{\text{stretched}}^{\text{set}}-$ (−11.48%)	$V_{\text{stretched}}^{\text{set}}+$ (10.18%)	$V_{\text{stretched}}^{\text{set}}+$ (17.06%)	$V_{\text{stretched}}^{\text{set}}-$ (−12.22%)
2	$V_{\text{stretched}}^{\text{set}}+$ (10.9%)	$V_{\text{stretched}}^{\text{set}}-$ (−7.07%)	$V_{\text{stretched}}^{\text{set}}-$ (−15.92%)	$V_{\text{stretched}}^{\text{set}}+$ (11.62%)
3	*R*_per_ − (−5.4%)	*E*_*e*_ − (−6.29%)	*C*_*v*,data_ − (6.81%)	*C*_*v*,data_ − (4.82%)
4	*R*_per_ + (5.05%)	*E*_*e*_ + (6.03%)	*t*_onset,RA_ + (6.01%)	*t*_onset,RA_ + (−4.11%)
5	*C*_*v*,data_ − (4.57%)	*τ*_2,LV_ − (3.86%)	*C*_*v*,data_ + (−5.54%)	*C*_*v*,data_ + (−4.1%)
6	*C*_*v*,data_ + (−3.85%)	*τ*_2,LV_ + (−3.77%)	$E_{\text{fw,RV}}^{\text{min}}-$ (−3.9%)	$E_{\text{fw,RV}}^{\text{min}}-$ (3.08%)
7	*t*_onset,RA_ + (−3.43%)	*C*_*v*,data_ − (3.73%)	$E_{\text{fw,RV}}^{\text{min}}+$ (3.59%)	$E_{\text{fw,RV}}^{\text{min}}+$ (−2.85%)
8	$E_{\text{fw,RV}}^{\text{min}}-$ (2.57%)	$E_{\text{fw,LV}}^{\text{max}}-$ (−3.12%)	$E_{\text{fw,RA}}^{\text{min}}-$ (−2.28%)	$E_{\text{fw,LV}}^{\text{min}}-$ (2.06%)
9	$E_{\text{fw,RV}}^{\text{min}}+$ (−2.38%)	*R*_per_ − (2.87%)	$E_{\text{fw,RA}}^{\text{min}}+$ (2.11%)	$E_{\text{fw,RV}}^{\text{max}}-$ (−2.02%)
10	$E_{\text{fw,LV}}^{\text{min}}-$ (1.86%)	*t*_onset,RA_ + (−2.83%)	$E_{\text{fw,RV}}^{\text{max}}-$ (1.85%)	$E_{\text{fw,LV}}^{\text{min}}+$ (−1.92%)
11	$E_{\text{fw,LV}}^{\text{min}}+$ (−1.73%)	*C*_*v*,data_ + (−2.71%)	$E_{\text{fw,RV}}^{\text{max}}+$ (−1.55%)	*t*_onset,LA_ + (−1.84%)
12	$E_{\text{fw,RV}}^{\text{max}}-$ (−1.71%)	$E_{\text{fw,RV}}^{\text{min}}-$ (2.38%)	*E*_0,puv_ − (−1.52%)	$E_{\text{fw,RV}}^{\text{max}}+$ (1.68%)
13	*t*_onset,LA_ + (−1.66%)	*R*_per_ + (−2.32%)	*R*_per_ − (1.44%)	*t*_onset,LA_ − (−1.63%)
14	$E_{\text{fw,RV}}^{\text{max}}+$ (1.43%)	$E_{\text{fw,LV}}^{\text{max}}+$ (2.31%)	*E*_0,puv_ + (1.38%)	*t*_onset,RA_ − (−1.5%)
15	*t*_onset,LA_ − (−1.41%)	$p_{0}^{\text{art}}+$ (2.24%)	*R*_per_ + (−1.36%)	*R*_per_ − (1.23%)
16	*t*_onset,RA_ − (−1.32%)	$E_{\text{fw,RV}}^{\text{min}}+$ (−2.03%)	$E_{\text{fw,RA}}^{\text{max}}-$ (1.18%)	*R*_per_ + (−1.18%)
17	$p_{0}^{\text{art}}-$ (−1.17%)	*E*_*c*_ − (−1.74%)	*t*_onset,LA_ − (1.1%)	$E_{\text{fw,RA}}^{\text{max}}-$ (−1.12%)
18	$p_{0}^{\text{art}}+$ (1.16%)	*E*_*c*_ + (1.65%)	$E_{\text{fw,RA}}^{\text{max}}+$ (−1.02%)	*E*_0,puv_ − (−1.11%)
19	*E*_0,puv_ − (−1.04%)	*K*_S,LV_ − (1.62%)	$c_{\text{ven}}^{\text{min}}-$ (0.99%)	$E_{\text{fw,RA}}^{\text{min}}-$ (1.01%)
20	$E_{\text{fw,RA}}^{\text{max}}-$ (−0.95%)	*K*_S,LV_ + (−1.59%)	*t*_onset,LA_ + (−0.93%)	*E*_0,puv_ + (1%)

## 4 Discussion

This section is devoted to a discussion of presented results and how they compare to clinically available observations, followed by an in depth discussion about local sensitivity analysis results addressing main determinants of assessed variables as well as the interaction of different vascular compartments. The section ends with a discussion on potential applications and future developments.

### 4.1 Model assessment in terms of haemodynamic variables

The performance of the model in terms of its capacity to reproduce a normal haemodynamic state can be assessed from results reported in Table 3. This table reports a selected set of model-predicted main haemodynamic variables, as well as references values found in the clinical literature. ADAVN is able to correctly reproduce the selected indexes regarding the functioning of the left heart, as well as those that are used to evaluate the coupling of the left heart and systemic circulation. The same agreement can be found for mean pressure values in different vascular districts, such as main arteries and veins, as well as for the venous system and for the intracranial compartment. Pressure waveform characteristics are assessed in terms of pulse wave velocities, as well as assessing indexes regarding pulsatility and changes of the pressure waveform as it travels through the arterial system. With the exception of the pulse pressure amplification index (PPA index in Table 3), all other evaluated indexes are in good agreement with clinical data. The low PPA index is due to the fact that in ADAVN we observe an amplification of the pulse pressure at the level of the brachial artery that is smaller than the one clinically observed, since the pulse pressure in the aortic root is within the physiological range (PP_A_ in Table 3). Furthermore, assessed model outputs regarding blood flow distribution are also aligned with reference data. In particular, cardiac output matches reference average values, as it does its distribution into the two vascular districtics on which this study focuses, i.e., the cerebral and coronary circulations. A closer look into main feeding arteries of these two vascular districts shows that these vessels receive blood amounts that are in agreement with average flows commonly measured in these vessels.

### 4.2 Model assessment in terms of haemodynamic waveforms

Figure 4 illustrates pressure and flow rate waveforms in sampled vessels, as well as their typical in vivo-acquired counterparts. Typical features that characterize arterial blood flow are the dicrotic notch in the aortic root (subfigure 4. A-I), and the pressure impingement when moving to peripheral districts, specifically the brachial-radial pathway (compare subfigures 4. A-I and 4. B-I). Arterial flow in central arteries is markedly systolic, with a waveform that changes significantly from the ascending to the abdominal aorta (compare subfigures 4. A-II and 4. B-II). The blood flow to the brain yields a characteristic flow rate at the common carotid artery, which is the consequence of the low-resistance cerebral territory, resulting in a relatively low diastolic flow rate (see subfigure 4. E-II). All these results are in agreement with data previously reported (Blanco et al., 2014; Blanco et al., 2015). Figure 4 also presents pressure and flow rate waveforms for selected venous vessels. The pressure waveforms feature the typical characteristics prescribed by the backward expansive and compressive waves generated by the right atrium contraction (see, for example, subfigure 4. C-I). In turn, the flow rate waveforms feature the typical bi-phasic V-notch after systole, more pronounced as we are closer to the right atrium such as in the superior vena cava, and diminished in intensity when we move to distal districts such as the jugular veins (compare subifgures 4. D-II and 4. F-II). Specific vessels corresponding to the head and neck circulation are also displayed in Figure 4. We find here a good qualitative agreement for both pressure and flow waveforms in the selected arteries and veins. Concerning the coronary circulation, in subfigures 4. G-I and 4. G-II, we can see how the model reproduces well the diastolic character of arterial coronary flow and the systolic pattern in venous coronary flow.

### 4.3 Sensitivity of main cardiovascular variables to model parameters

Table 4 reports results on local sensitivity of mean arterial pressure (MAP), aortic pulse pressure (PP_A_), central venous pressure (CVP) and cardiac output (CO) to the 20 most influential parameters according to our local sensitivity analysis study.

#### 4.3.1 On relevance of parameters

Considering the ranking position of parameters for all variables of interest, it is remarkable to observe how the total stretched volume ( $V_{\text{stretched}}^{\text{set}}$ in Table 4) is the most influential parameter in all cases. Noteworthy, its contribution is not only ranking first, but its impact on considered variables is 2–3 times higher than the impact of second-ranked parameters. Another parameter that ranks high for most variables is the total venous compliance (*C*_*v*,data_ in Table 4). This parameter either ranks second or is placed third to fourth for all variables, with an impact very similar to the one of preceding parameters in terms of caused percentage change.

#### 4.3.2 Determinants of MAP

This variable, as all others considered here, is mostly influenced by the total streched volume. In our model this variable is directly linked to the total blood volume since the unstressed volume is assumed fixed and constant in time. Interestingly, while the second-ranked parameter is the total peripheral resistance, which regards directly the systemic circulation, the third-to fourth-ranked parameters regard the venous circulation and the right heart. This result evidences how a variable normally considered as mainly characterized by the arterial side of circulation is also strongly influenced by properties of the venous side, as well as of the right heart, with a relevant role played by the timing of the right atrium. Notably, the first parameter regarding the tube law used for arteries that appears in our rank of most influential parameters for MAP is the reference pressure $p_{0}^{\text{art}}$ , appearing in tube law (Eq. 3), which ranks 17-th.

#### 4.3.3 Determinants of PP_A_

In this case the impact of total stretched volume is less pronounced than for other variables, but still almost twice that of the next parameter, which in this case is the reference elastine Young modulus (*E*_*e*_). This index shows a stronger dependence on arterial parameters, such as the relaxation time *τ*_2,LV_ and the maximum free wall elastance of the left ventricle $E_{\text{fw},LV}^{\text{max}}$ . Interestingly, also in this case the venous system is contributing with an impact similar to that of the two mentioned parameters.

#### 4.3.4 Determinants of CVP

The leading role of total stretched volume is also found here, where it also has the largest impact in terms of percentage change with respect to the nominal CVP value. The next parameters in the ranking are always related to the venous system state and the right heart function. Remarkably, a parameter regarding pulmonary circulation, the reference-volume elastance of pulmonary veins, *E*_0,puv_, ranks higher than arterial parameters.

#### 4.3.5 Determinants of CO

As for all other variables, also in this case the leading role is played by total stretched blood volume. Then, the most influential parameters regard either the systemic veins or the functioning of the right heart, evidencing how the role played by parameters regarding the left heart and the systemic arterial circulation is less relevant than that played by other cardiovascular components in determining this fundamental haemodynamic quantity.

#### 4.3.6 Interaction between arterial and venous circulation

Results reported in Table 4 put in evidence the strong connection between the arterial and venous districts. In fact, parameters regarding the venous circulation or the right heart rank always high in terms of sensitivity of *arterial* variables to model parameters. When considering CVP the connection is less evident, with the first parameter related to the arterial system, total peripheral resistance, ranking only 12-th for this variable. However, the connection becomes more evident in a variable that can be considered as indicative of the state on both, the arterial and the venous compartments, namely, the cardiac output CO, which is influenced predominantly by venous circulation and right heart parameters, but where the contribution of left heart and arterial parameters is more pronounced. This strong connection should be kept in mind by modellers when addressing modelling questions in which disregarding the interplay between these two major districts of circulation might result in a strongly biased description of the actual physiological processes intended to be described.

## 5 Concluding remarks and future work

In this work we described the construction of the first version of the ADAVN model, which combines the most complete existing arterial model, with a novel venous network model, featuring a detailed description of cerebral and coronary venous compartments. Being this the first communication on ADAVN, we have decided to focus on a detailed description of the model components and parameters, aiming at reproducibility of published results. In fact, we provide all necessary parameters and data for the construction of the model, including the full description of network connectivity and vessels’ geometry. Having this goal in mind, we have limited the content regarding modelling results to a general validation of model outputs with respect to clinical reference data and waveforms and to a local sensitivity analysis. This set of results allowed us to discuss the capacity of the model to produce physiologically sound results, as well as to gain knowledge on the relevance of model parameters and, more importantly, model districts, in the determination of global haemodynamic variables. A significant output of the presented sensitivity analysis is the identification of a great influence of properties of the venous district in the determination of main cardiovascular variables. This aspect should be carefully considered by modellers addressing pathological states in which the venous system is expected to undergo changes with respect to a physiological condition, since the impact of those changes might be dominant over the ones of other vascular districts.

The ADAVN model is a natural evolution of the ADAN model (Blanco et al., 2015) and is intended to be the backbone on which to incrementally add model components regarding physiological aspects of cardiovascular physiology, as well as model components rearding the interaction of blood with other solid and fluid compartments such as intracranial tissues and fluids, the respiratory system, and the lymphatic system. Similar models to ADAVN, but of reduced complexity in terms of the level of detail of vascular networks, have been already developed by the authors of this manuscript to address some of the above named applications (Müller and Toro, 2014a; Celant et al., 2021; Toro et al., 2022). The distinctive and unique aspect of ADAVN regarding the fact that vessels are described not only in terms of connectivity, length and radius, but also by information on their three-dimensional position, makes ADAVN an excellent framework to model orthostatic stress, as well as to explore the role that the extension of currently available models, as, for example, an assessment of the impact that including the curvature in 1D vessels, especially at junctions, might have on model outputs. The ADAVN model allows to explore the impact of modelling hypotheses on a wide range of spatial scales involving the arterial and the venous districts.

## Data Availability

The original contributions presented in the study are included in the article/Supplementary Material, further inquiries can be directed to the corresponding author.
