# Supplementary material for: An anatomically detailed arterial-venous network model. Cerebral and coronary circulation
Source: Front Physiol. 2023 Jun 26;14:1162391. doi: 10.3389/fphys.2023.1162391 (PMC10332167; doi:10.3389/fphys.2023.1162391)
Supplement: Supplementary file 1 [file DataSheet2.pdf]

Supplementary Material

APPENDIX

A Model parameters

Next, we provide the data required to define all model parameters. These data are complementary to the Supplementary File adavn_vessels.csv.

Parameter	Units	RA	RV	LA	LV
$V_{0,\alpha}$	cm^3	7	20	3	10
$V_\alpha(0)$	cm^3	67	172	71	136
$\mu_{AV,\alpha}$	-	2	3	4	3
$t_{\text{onset},\alpha}/T$	-	0.8125	0.	0.8125	0
$\tau_{1,\alpha}/T$	-	0.0525	0.2687	0.0525	0.2687
$m_{1,\alpha}$	-	1.99	1.32	1.99	1.32
$\tau_{2,\alpha}/T$	-	0.1725	3	0.1725	3
$m_{2,\alpha}$	-	11.2	21.9	11.2	21.9
$E^{\text{min}}_{\text{fw},\alpha}$	dyn/cm^5	60	65	120	93
$E^{\text{max}}_{\text{fw},\alpha}$	dyn/cm^5	150	800	210	4476
$\mathcal{K}_{\text{C}}$	-	2	6	2	6
$K_{\text{S},\alpha} \times 10^{-3}$	s/cm^3	0.5	1.0	0.25	0.5

Table S1. Parameters used for models of the four cardiac chambers presented in Section 2.2.2. T is the cardiac cycle duration, set to $T = 0.8s$. RA, RV, LA and LV stand for right/left atrium/ventricle. $V_\alpha(0)$ is the chamber volume assigned as initial condition. Parameters are modified from Mynard and Smolich (2015).

Parameter	Units	Value
K_{pc}	dyn/cm^2	666.61184
$V_{0,\text{pc}}$	cm^3	700
Φ_{pc}	cm^3	40
V_{mio}	cm^3	192
V_{pcf}	cm^3	30

Table S2. Parameters used for the pericardium pressure model presented in Section 2.2.2. Parameters are modified from Suga et al. (1973).

Parameter	Units	TV	PV	MV	AV
$l_{\text{eff},\beta}$	cm	2	1.5	2	1
$A^{\text{max}}_{\text{eff},\beta}$	cm^2	6	5.7	5.1	4.9
$A^{\text{min}}_{\text{eff},\beta}$	cm^2	10^{-5}	10^{-5}	10^{-5}	10^{-5}
$K_{\text{vo},\beta}$	cm^2/dyn	0.03	0.02	0.02	0.02
$K_{\text{vc},\beta}$	cm^2/dyn	0.04	0.02	0.04	0.02
$\Delta p_{\text{open},\beta}$	dyn/cm^2	0	0	0	0
$\Delta p_{\text{close},\beta}$	dyn/cm^2	0	0	0	0

Table S3. Parameters used for models of the four cardiac valves presented in Section 2.2.2. TV, PV, MV and AV stand for tricuspid, pulmonary, mitral and aortic valves. Parameters are modified from Mynard and Smolich (2015).

Parameter	Units	pua	puc	puv
$E_{0,\gamma}$	dyn/cm^5	26.66	26.66	26.66
$V_{0,\gamma}$	cm^3	20	60	200
$p_\gamma(0)$	dyn/cm^2	10^4	10^4	10^4
Ω_γ	$g/s/cm^4$	13.33	13.33	13.33
L_γ	dyn/cm^5s^2	0.67	0.67	0.67
R_γ	dyn/cm^5s	53.33	53.33	6.67

Table S4. Parameters used for the pulmonary circulation model presented in Section 2.2.3. pua, puc and puv stand for arterial, capillary and venous pulmonary compartments. Parameters are modified from Suga et al. (1973).

Vessel	Chamber	Region	Per. vol. [cm^3]	Tot. resist. [dyn/cm^5s]
1566	LV	LVFW	23.12	112967.81
1571	LV	LVFW	17.46	148252.62
1574	IVS	IVS	9.36	276144.04
1576	LV	LVFW	41.03	72904.73
2907	LV	LVFW	29.81	92067.83
2898	RV	RVFW	5.60	369431.43
2905	RV	RVFW	13.73	170345.90
2906	RV	RVFW	17.10	157229.87
1564	LV/RV	IVS	13.58	198182.16
1565	LV/RV	IVS	13.58	202278.80
2901	LV/RV	IVS	5.16	510107.84
2902	LV/RV	IVS	7.06	363522.56
1572	LV/RV	IVS	9.36	274140.99
1575	LA	LAW	13.76	229003.90
2897	RA	RAW	8.43	595208.85
2899	RV	RVFW	5.60	449487.43
2900	RA	RAW	7.05	694693.68
2908	RV	RVFW	5.60	416802.86

Table S5. Indexes of terminal coronary arteries, chamber acting on peripheral bed, region of myocardium perfused, perfused myocardial volumes and total peripheral resistances for models of the coronary peripheral beds presented in Section 2.2.5. Vessel index is related to the ordering shown in the Supplementary File adavn¯vessels.csv. LV: left ventricle; RV: right ventricle; LA: left atrium; RA: right atrium; LVFW: left ventricle free wall; RVFW: right ventricle free wall; IVS: intra-ventricular septum; LAW: left atrium wall; RAW: right atrium wall. Values computed as described in Section 2.4.6

Code	Name
1	head
2	encephalon
3	brain
4	cerebellum
5	pons
6	duramater
7	scalp
8	face
9	neck
10	right upper limb
11	right shoulder
12	right arm
13	right forearm
14	right hand
15	left upper limb
16	left shoulder
17	left arm
18	left forearm
19	left hand
20	trunk
21	aorta
22	aortic arch
23	thoracic aorta
24	abdominal aorta
25	chest
26	coronaries
27	right coronaries
28	left coronaries
29	sternum
30	abdomen

Code	Name
31	diaphragm
32	right suprarenal
33	left suprarenal
34	liver
35	stomach
36	spleen
37	pancreas
38	right kidney
39	left kidney
40	intestine
41	dorsum
42	spinal
43	spinal cord
44	cervical spinal cord
45	thoracic spinal cord
46	lumbar spinal cord
47	lumbar
48	cauda equina
49	pelvis
50	right lower limb
51	right gluteal region
52	right hip tight
53	right knee
54	right leg
55	right foot
56	left lower limb
57	left gluteal region
58	left hip tight
59	left knee
60	left leg
61	left foot

Table S6. Region codes used to determine in which part of the body vessels are located.

Valve index	Upstream	Downstream
1	4320 (-1)	4057 (1)
2	4321 (-1)	4196 (1)
3	4322 (-1)	4055 (1)
4	4323 (-1)	4194 (1)
5	4324 (-1)	4087 (1)
6	4325 (-1)	4223 (1)
7	4326 (-1)	4284 (1)
8	4327 (-1)	4286 (1)
9	4328 (-1)	4306 (1)
10	4329 (-1)	4292 (1)
11	4330 (-1)	4176 (1)
12	4331 (-1)	4177 (1)
13	4332 (-1)	4164 (1)
14	4333 (-1)	4165 (1)
15	4334 (-1)	4182 (1)
16	4335 (-1)	4170 (1)
17	4336 (-1)	4183 (1)
18	4337 (-1)	4171 (1)
19	4338 (-1)	4173 (1)
20	4339 (-1)	4174 (1)
21	4340 (-1)	4161 (1)
22	4341 (-1)	4162 (1)
23	4342 (-1)	4180 (1)
24	4343 (-1)	4168 (1)
25	4344 (-1)	4179 (1)
26	4345 (-1)	4181 (1)
27	4346 (-1)	4167 (1)
28	4347 (-1)	4169 (1)
29	4348 (-1)	4145 (1)
30	4262 (1)	4349 (-1)

Table S7. List of venous valves present in the ADAVN model. Upstream and downstream vessel index. Orientation of vessel with respect to valve in brackets: 1 for vessel sharing an outlet node, -1 for vessel sharing an inlet node.

Starling resistor index	Upstream	Downstream
1	4350 (-1)	4043 (1)
2	4044 (1)	4351 (-1)
3	4075 (1)	4352 (-1)
4	4353 (-1)	4083 (1)
5	4085 (1)	4354 (-1)
6	4086 (1)	4355 (-1)
7	4356 (-1)	4092 (1)
8	4357 (-1)	4093 (1)
9	4358 (-1)	4100 (1)
10	4359 (-1)	4101 (1)
11	4360 (-1)	4102 (1)
12	4361 (-1)	4103 (1)
13	4362 (-1)	4104 (1)
14	4363 (-1)	4105 (1)
15	4364 (-1)	4106 (1)
16	4365 (-1)	4107 (1)
17	4366 (-1)	4108 (1)
18	4367 (-1)	4109 (1)
19	4368 (-1)	4111 (1)
20	4369 (-1)	4112 (1)
21	4370 (-1)	4113 (1)
22	4371 (-1)	4114 (1)
23	4372 (-1)	4115 (1)
24	4373 (-1)	4116 (1)
25	4374 (-1)	4118 (1)
26	4375 (-1)	4139 (1)
27	4376 (-1)	4140 (1)
28	4377 (-1)	4141 (1)
29	4378 (-1)	4186 (1)
30	4187 (1)	4379 (-1)
31	4213 (1)	4380 (-1)
32	4381 (-1)	4221 (1)
33	4222 (1)	4382 (-1)
34	4383 (-1)	4228 (1)
35	4384 (-1)	4229 (1)
36	4385 (-1)	4236 (1)
37	4386 (-1)	4237 (1)
38	4387 (-1)	4238 (1)
39	4388 (-1)	4239 (1)
40	4389 (-1)	4240 (1)
41	4390 (-1)	4241 (1)
42	4391 (-1)	4242 (1)
43	4392 (-1)	4243 (1)
44	4393 (-1)	4244 (1)
45	4394 (-1)	4245 (1)
46	4395 (-1)	4247 (1)
47	4396 (-1)	4248 (1)
48	4397 (-1)	4249 (1)
49	4398 (-1)	4250 (1)
50	4399 (-1)	4251 (1)
51	4400 (-1)	4252 (1)
52	4401 (-1)	4254 (1)
53	4402 (-1)	4258 (1)

Table S8. List of Starling resistors present in the ADAVN model. Upstream and downstream vessel index. Orientation of vessel with respect to valve in brackets: 1 for vessel sharing an outlet node, -1 for vessel sharing an inlet node.

B Definition of cardiac and cardiovascular indexes

We provide here a detailed explanation of how indexes presented in Table 3 were computed:

- LVSV (left ventricle stroke volume):

$$\mathrm{LVSV} = \mathrm{LVEDV} - \mathrm{LVESV}\,,$$

  with LVEDV and LVESV being left ventricle end diastolic and systolic volumes.
- LVEF (left ventricle ejection fraction):

$$\mathrm{LVEF} = \frac{\mathrm{LVSV}}{\mathrm{LVEDV}}\,.$$

- $\mathrm{E_{LV}I}$ (left ventricle elastance index):

$$\mathrm{E_{LV}I} = \frac{\mathrm{ESP}}{\mathrm{LVESV}} \times \mathrm{BSA}\,,$$

  where ESP is end systolic brachial artery blood pressure (vessel 2672) and BSA is the body surface area, taken equal to 1.6488 m^2 Blanco et al. (2014).
- EaI (arterial elastance index):

$$\mathrm{EaI} = \frac{\mathrm{ESP}}{\mathrm{LVSV}} \times \mathrm{BSA}\,,$$

- MAP/SBP/DBP (mean/systolic/diastolic blood pressure): cardiac cycle-averaged, maximum and minimum central pressure (vessel 816).
- MPAP (mean pulmonary arterial pressure): cardiac cycle-averaged pressure in the arterial pulmonary compartment.
- ICP (intracranial pressure): cardiac cycle-averaged intracranial pressure p_{ICP}.
- CVP (central venous pressure): cardiac cycle-averaged pressure in the right atrium.
- $\mathrm{PWV_{CF}}$: carotid-femoral pulse wave velocity computed between the right common carotid artery (vessel 736) and the left femoral artery (vessel 2437) with timing computed based on foot identification at intersection of horizontal line of minimum pressure and tangent at maximum pressure slope Millasseau et al. (2005).
- $\mathrm{PWV_{FP}}$: femoral-posterior tibial pulse wave velocity computed between the left femoral artery (vessel 2437) and the left posterior tibial artery (vessel 2198) with timing computed based on foot identification at intersection of horizontal line of minimum pressure and tangent at maximum pressure slope Millasseau et al. (2005).
- ABI (ankle-brachial index): computed as ratio of systolic pressure between the left posterior tibial artery (vessel 2198) and the left brachial artery (vessel 1362).
- PPA (pulse pressure amplification): computed as the pulse pressure ratio between the brachial artery (vessel 1362) and the ascending aorta (vessel 818).
- $\mathrm{PP_A}$ (pulse pressure in the ascending aorta): computed as the pulse pressure in vessel 818.
- $\mathrm{PP_F}$ (pulse pressure in the left femoral artery): computed as the pulse pressure in vessel 2436.

REFERENCES

Blanco, P., Watanabe, S., Dari, E., Passos, M., and Feijóo, R. (2014). Blood flow distribution in an anatomically detailed arterial network model: criteria and algorithm. *Biomechanics and Modeling in Mechanobiology* 13, 1303–1330

Millasseau, S. C., Stewart, A. D., Patel, S. J., Redwood, S. R., and Chowienczyk, P. J. (2005). Evaluation of Carotid-Femoral Pulse Wave Velocity: Influence of Timing Algorithm and Heart Rate. *Hypertension* 45, 222–226. doi:10.1161/01.HYP.0000154229.97341.d2

Mynard, J. and Smolich, J. (2015). One-dimensional haemodynamic modeling and wave dynamics in the entire adult circulation. *Annals of Biomedical Engineering* 43, 1443–1460

Suga, H., Sagawa, K., and Shoukas, A. A. (1973). Load independence of the instantaneous pressure-volume ratio of the canine left ventricle and effects of epinephrine and heart rate on the ratio 32, 314–322
